# SARS-CoV-2-specific T cells in unexposed adults display broad trafficking potential and cross-react with commensal antigens

**DOI:** 10.1126/sciimmunol.abn3127

**Published:** 2022-07-14

**Authors:** Laurent Bartolo, Sumbul Afroz, Yi-Gen Pan, Ruozhang Xu, Lea Williams, Chin-Fang Lin, Ceylan Tanes, Kyle Bittinger, Elliot S. Friedman, Phyllis A. Gimotty, Gary D. Wu, Laura F. Su

**Affiliations:** ^1^ Department of Medicine, Division of Rheumatology, Perelman School of Medicine, Institute for Immunology, University of Pennsylvania, Philadelphia, PA 19104, USA.; ^2^ Corporal Michael J Crescenz VA Medical Center, Philadelphia, PA, 19104, USA.; ^3^ Division of Gastroenterology and Hepatology, Perelman School of Medicine, University of Pennsylvania, Philadelphia, PA 19104, USA.; ^4^ Department of Biostatistics, Epidemiology, and Informatics, Perelman School of Medicine, University of Pennsylvania, Philadelphia PA 19104, USA.; ^5^ Division of Gastroenterology, Hepatology and Nutrition, Children’s Hospital of Philadelphia, PA, 19104, USA.

## Abstract

The baseline composition of T cells directly impacts later response to pathogens, but the complexity of precursor states remains poorly defined. Here, we examined the baseline state of SARS-CoV-2-specific T cells in unexposed individuals. SARS-CoV-2-specific CD4^+^ T cells were identified in pre-pandemic blood samples by class II peptide-MHC tetramer staining and enrichment. Our data revealed a substantial number of SARS-CoV-2-specific T cells that expressed memory phenotype markers. Integrated phenotypic analyses demonstrated diverse pre-existing memory states that included cells with distinct polarization states and trafficking potential to barrier tissues. T cell clones generated from tetramer-labeled cells cross-reacted with antigens from commensal bacteria in the skin and gastrointestinal tract. Direct ex vivo tetramer staining for one spike-specific population showed a similar level of cross-reactivity to sequences from endemic coronavirus and commensal bacteria. These data highlight the complexity of precursor T cell repertoire and implicate non-infectious exposures to common microbes as a key factor that shapes human pre-existing immunity to SARS-CoV-2.

## Teaser

Pre-existing immunity to SARS-CoV-2 displayed diverse phenotypes, had broad tissue tropism and cross-reacted with commensal antigens.

## INTRODUCTION

SARS-CoV-2 infection (COVID-19) presents with a myriad of organ involvement, variable duration of symptoms, and diverse clinical presentations that range from asymptotic infection to death (*1*–*6*). The underlying determinants for variable responses to COVID-19 and other infections remain incompletely understood. Age, sex, co-morbidities, and host genetics have emerged as key factors that increase the risk for severe COVID-19 (*7*–*12*). Studies in mice have additionally demonstrated a key role of environment on pathogen resistance, with an accumulation of memory T cells that modulate host protection in mice raised in a free-living environment (*13*, *14*). What remains less clear is how the environment shapes human immune responses to SARS-CoV-2 and other pathogens.

Prior studies have identified T cells that recognize SARS-CoV-2 in unexposed individuals, including cells that expressed an antigen-experienced phenotype (*15*–*22*). The identification of SARS-CoV-2-specific memory cells with presumed functional superiority against infection has led to excitement over the source of antigens that drive these types of responses (*23*). A widely held view is that pre-existing memory to SARS-CoV-2 reflects past exposures to common circulating coronaviruses (CCCoV) (*24*). Consistent with this, short-term lines expanded by SARS-CoV-2 peptides identified T cells that responded to analogous sequences from CCCoV (*16*, *25*). However, other studies found weak T cell cross-reactivity between SARS-CoV-2 and CCCoV (*22*, *26*). The existence of other potential sources was suggested by a sequence conservation analysis, which did not find higher proportion of matches between SARS-CoV-2 and other sequenced members of *Coronaviridae* family than expected by chance (*27*). Furthermore, pre-existing memory is not unique to SARS-CoV-2. We and others have identified pre-existing memory T cells that recognized other pathogens, including yellow fever virus (YFV) that does not circulate in the United States and lacks a common close relative to simulate prior exposure (*28*–*31*).The acquisition of a memory phenotype in the absence of similar pathogens suggest other avenues for acquiring an antigen-experienced state at baseline.

Here, we performed an in-depth analysis of SARS-CoV-2-specific T cells in unexposed individuals and investigated alternative sources of antigens that could have driven this pre-existing T cell differentiation. Rare SARS-CoV-2-specific CD4^+^ T cells were identified directly ex vivo using class II peptide-MHC (pMHC) tetramer enrichment. Blood samples collected prior to year 2020 were used to ensure the absence of prior SARS-CoV-2 exposure. The activation of naïve T cells in an anatomical niche imprints selective trafficking properties (*32*). For example, lamina propria-derived dendritic cells (DCs) in the mesenteric lymph nodes efficiently induce gut-tropic receptors, CCR9 and α4β7 (*33*–*36*). Conversely, cutaneous DCs imprint CCR10 expression to enable T cells primed in the skin to traffic back to the cutaneous tissue (*37*). Hypothesizing that the imprints of initial antigen engagement are retained in pre-existing T cells, we used trafficking molecules to gain insights into where prior antigen engagement might have occurred. We showed that pre-existing SARS-CoV-2-specific memory CD4^+^ T cells expressed diverse phenotypic markers of various CD4^+^ T cell lineages, displayed gut and skin tropism, and cross-reacted with commensal bacteria. Cross-reactivity to bacterial antigens was identified for multiple SARS-CoV-2 epitopes and occurred at a similar overall frequency as cross-reactive responses to homologous sequences from CCCoV for a spike-specific population. These findings highlight the breadth of SARS-CoV-2-specific T cells and implicate non-infectious microbial stimuli as a major factor that guides pre-existing immune responses to SARS-CoV-2.

## RESULTS

### Pre-existing SARS-CoV-2-specific T cells in unexposed adults

**Fig. 1.**
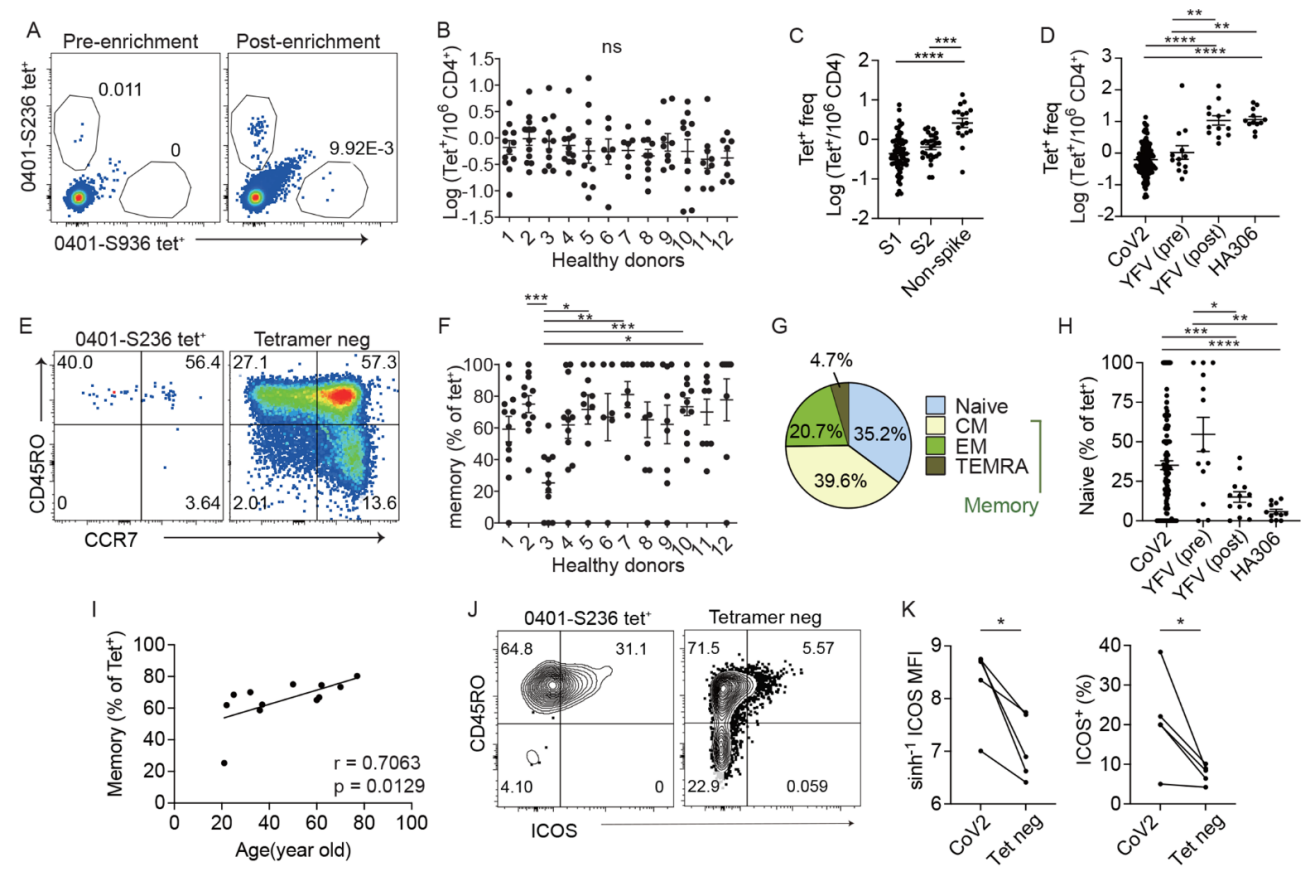
Identification of SARS-CoV-2-specific T cells in unexposed individuals. (A) Direct ex vivo staining, pre- and post-magnetic enrichment, of representative SARS-CoV-2 tetramer^+^ cells using cryopreserved cells obtained before year 2020. Plots represent one of two independent experiments. (B) The frequency of SARS-CoV-2-specific CD4^+^ T cells across 12 unexposed healthy donors by direct ex vivo tetramer staining. Each symbol represents data from a SARS-CoV-2-specific population repeated 2.04 times (±0.07). (C) Plot summarizes the frequencies of T cells that recognized different regions of SARS-CoV-2. (D) Frequency of T cells that recognized epitopes from SARS-CoV-2, YFV, or influenza virus. Plot combines 12 SARS-CoV-2-specific populations and HA306 tetramer^+^ cells from 12 donors. YFV-specific T cells before (pre) or after (post) vaccination include 11 YFV-specific populations from HD1 and HD2. (E) Plots show CD45RO and CCR7 staining to divide cells into naïve or memory subsets. Plots represent one of two independent experiments. (F) The abundance of pre-existing memory T cells as a percentage of each tetramer^+^ population shown in B. (G) The averaged differentiation phenotype of all SARS-CoV-2 tetramer^+^ cells examined: naïve (CD45RO^-^CCR7^+^), central memory (CM, CD45RO^+^CCR7^+^), effector memory (EM, CD45RO^+^CCR7^-^), and TEMRA (CD45RO^-^CCR7^-^). (H) The proportion of naïve cells as a percentage of tetramer^+^ cells shown in D. (I) Correlation between the abundance of memory precursors and donor age. Distinct tetramer^+^ populations from the same donor were combined and represented as an average (n = 12). (J) ICOS staining on a representative tetramer^+^ population and tetramer^-^ CD4^+^ T cells. Plots represents one of two independent experiments. (K) ICOS expression by median staining intensity (MFI, left) or as a percentage (right) of SARS-CoV-2 (CoV2) or total CD4^+^ T cells. Each symbol represents data repeated 1.59 times (±0.17). Line connects averaged data from the same donor (n = 5). (B, C, D, F, and H) used Welch’s ANOVA. P-values for pairwise comparisons were computed using Dunnett’s T3 procedure. For (I), Spearman correlation was computed. Line represents least square regression line. For (K), a paired *t* test was performed. Mean ± SEM. * p < 0.05, ** p < 0.01, *** p < 0.001. **** P < 0.0001.

We analyzed PBMC from twelve unexposed healthy donors (HD) using pre-pandemic blood samples collected prior to 2020 (Table S1). SARS-CoV-2-specific CD4^+^T cells were identified using a direct ex vivo approach with pMHC tetramers. We recombinantly expressed HLA-DRA/DRB1*0401 monomers (DR4). A set of 12 peptides that stimulated T cells in COVID-19 patients based on prior studies and predicted to bind HLA-DR4 were selected for generation of tetramers (Table S2). We coupled tetramer staining with magnetic column-based enrichment to enable enumeration of rare tetramer-labeled T cells in the unprimed repertoire. The baseline differentiation states of tetramer-labeled T cells were delineated by anti-CD45RO and CCR7 antibody staining. In total, we analyzed 117 SARS-CoV-2-specific CD4^+^ populations in 12 healthy unexposed adults. SARS-CoV-2 precursors varied by epitope-specificity and showed heterogeneity between individuals (Fig. 1A-B, Fig. S1A-B). On average, the frequency of spike-specific T cells was lower compared to ORF8 and ORF9-specific T cells that recognized epitopes outside of the spike region (Fig. 1C). Next, we compared SARS-CoV-2-specific T cells to other antigen-naïve or experienced populations (Table S3). Two of the study volunteers had previously participated in a yellow fever virus (YFV) vaccine study and had YFV-specific CD4^+^ T cells analyzed before and after vaccination (*30*). Because most people likely have been exposed to influenza virus by vaccination and/or infection, we also stained for T cells that recognized hemagglutinin antigen (HA) of influenza virus as another antigen-experienced population. Consistent with an antigen inexperienced state, the overall frequency of SARS-CoV-2-specific T cells was similar to that of YFV-specific T cells in unexposed individuals and significantly lower than HA-specific T cells or YFV-specific T cells after vaccination (Fig. 1D, Fig. S2).

To examine the differentiation state of SARS-CoV-2-specific pre-immune T cells, we broadly divided tetramer-labeled cells by CD45RO and CCR7 staining. This revealed heterogeneous memory phenotypes that differed by epitope specificity and varied across donors (Fig. 1E-F, S1C). Collectively, only 35.2% of SARS-CoV-2-specific precursors were naïve. The remaining cells expressed a memory phenotype, which included 39.6% central memory cells (CM), 20.7% effector memory cells (EM), and 4.7% terminal effector cells (TEMRA) (Fig. 1G). While naïve SARS-CoV-2-specific T cells comprised of a minor subset, they were still substantially more abundant than the naïve component of post-exposure T cells that recognized HA or YFV (Fig. 1H). Interestingly, SARS-CoV-2-specific T cells contained a slightly smaller naïve fraction compared to YFV-specific T cells in unexposed individuals (Fig. 1H). Previous exposures to endemic coronaviruses may contribute to this difference, although the abundance of pre-existing memory cells did not correlate with the conservation of SARS-CoV-2 epitopes with CCCoV sequences (Fig. S1D, Table S4). Beyond CCCoV, past studies show a relationship between the proportion of memory cells within SARS-CoV-2-specific T cells and the overall immunological experience of the donor (*22*). Consistent with this, the abundance of pre-existing SARS-CoV-2-specific memory T cells positively correlated with donor age (Fig. 1I) and showed a weak association with total memory cell frequency in our dataset (Fig. S1E). To investigate if antigen stimulation was occurring on an ongoing basis, we used ICOS as an activation marker to analyze T cell activation state (*30*, *38*). This showed higher ICOS expression on SARS-CoV-2-specific T cells compared to total CD4^+^ cells, suggesting that some pre-existing cells perceived stimulation signals at baseline (Fig. 1J-K). Collectively, direct ex vivo tetramer staining provided a quantification of SARS-CoV-2-specific precursors and identified pre-existing memory T cells in unexposed individuals. Baseline up-regulation of activation markers further suggested recognition of antigens beyond conserved epitopes from other coronaviruses.

### Pre-existing SARS-CoV-2-specific T cells expressed gut homing markers

**Fig. 2.**
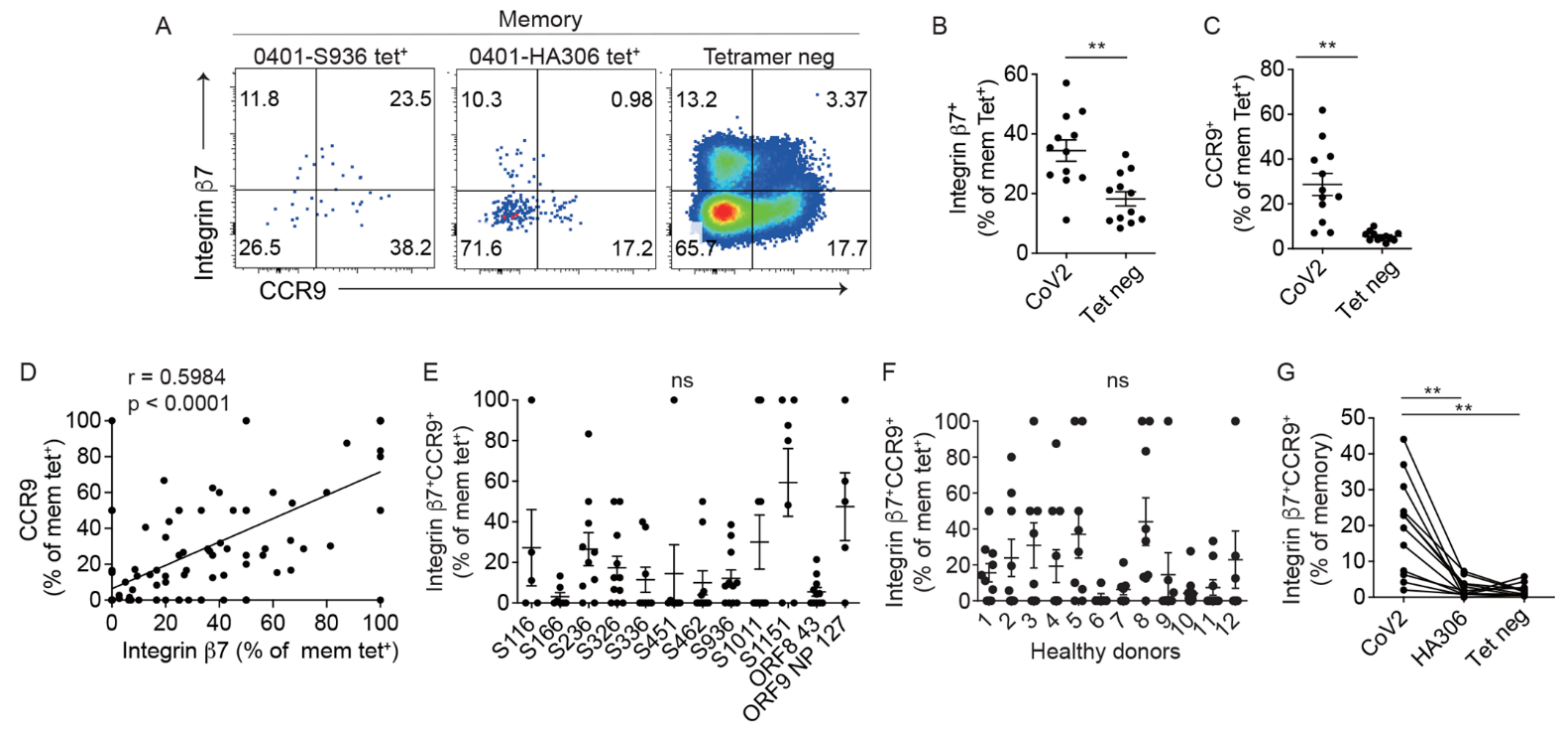
SARS-CoV-2-specific T cells express gut-trafficking receptors. (A) Representative integrin β7 and CCR9 staining on tetramer-labeled and background CD4^+^ T cells. Naïve cells were excluded. Plots represent one of four independent experiments. (B-C) Plot summarizes the proportion of integrin β7^+^ (B) and CCR9^+^ (C) cells as a percentage of SARS-CoV-2 (CoV2) tetramer^+^ or tetramer negative CD4^+^ memory cells. Each symbol represents data repeated 1.81 times (±0.11). Distinct tetramer^+^ populations from the same donor were combined and represented as an average. (D) The relationship between integrin β7 and CCR9 expression on SARS-CoV-2 tetramer^+^ memory cells. Each symbol represents data from a distinct SARS-CoV-2-specific population. (E-F) The abundance of integrin β7^+^CCR9^+^ cells as a percentage of SARS-CoV-2 tetramer^+^ memory cells by specificity (E) or donor (F). (G) Integrin β7^+^CCR9^+^ expression as a percentage of memory cells that recognized a SARS-CoV-2 peptide, HA306 peptide, or in the tetramer negative fraction. SARS-CoV-2 tetramer^+^ populations from the same donor were combined and represented as an average. Line connects data from the same donor (n = 12). For (B-C), a paired *t* test was performed. For (D), Spearman correlation was computed. Line represents least square regression line. (E) and (F) used Welch’s ANOVA. P-values for pairwise comparisons were computed using Dunnett’s T3 procedure. For (G), repeated measure one-way ANOVA was used and p-values for pairwise comparisons were computed using Tukey’s multiple comparison test. Mean ± SEM. ** p < 0.01.

The intestinal tract is home to trillions of microbial organisms (*39*). The composition of the microbiome is heavily dependent on the living environment and has a critical impact on human health (*13*, *40*). Additionally, previous studies demonstrate that homeostatic interactions with microbes in the gut are sufficient to drive human memory T cell differentiation (*41*). We therefore hypothesized that the gut-microbiome could be a source of ongoing antigenic stimulation that shapes the pre-existing SARS-CoV-2-specific T cell repertoire. Flexible engagement of TCRs may then allow some commensal-activated T cells to be detected as memory cells to a new pathogen. To begin to test this idea, we used gut homing receptors, integrin β7 and CCR9, to infer priming by and/or the potential to engage intestinal antigens (*35*, *42*, *43*). Tetramer staining for SARS-CoV-2-specific T cells was combined with antibody staining for trafficking receptors. HA-specific T cells were identified for comparison. This revealed a higher proportion of SARS-CoV-2-specific integrin β7^+^ or CCR9^+^ cells compared to the background level on tetramer negative memory CD4^+^ T cells (Fig. 2A-C). SARS-CoV-2-specific T cells co-expressed CCR9 and Integrin β7 (Fig. 2D), which showed a variable pattern but as a group was more abundant within SARS-CoV-2 tetramer^+^ populations compared to influenza-specific T cells or total memory cells (Fig. 2E-G). Taken together, these data support the possibility that chance encounters with antigens in the intestinal environment could promote differentiation of T cells that recognize an unrelated pathogen.

### Pre-existing SARS-CoV-2 tetramer^+^ cells expressed a variety of trafficking receptors and differentiation phenotypes

**Fig. 3.**
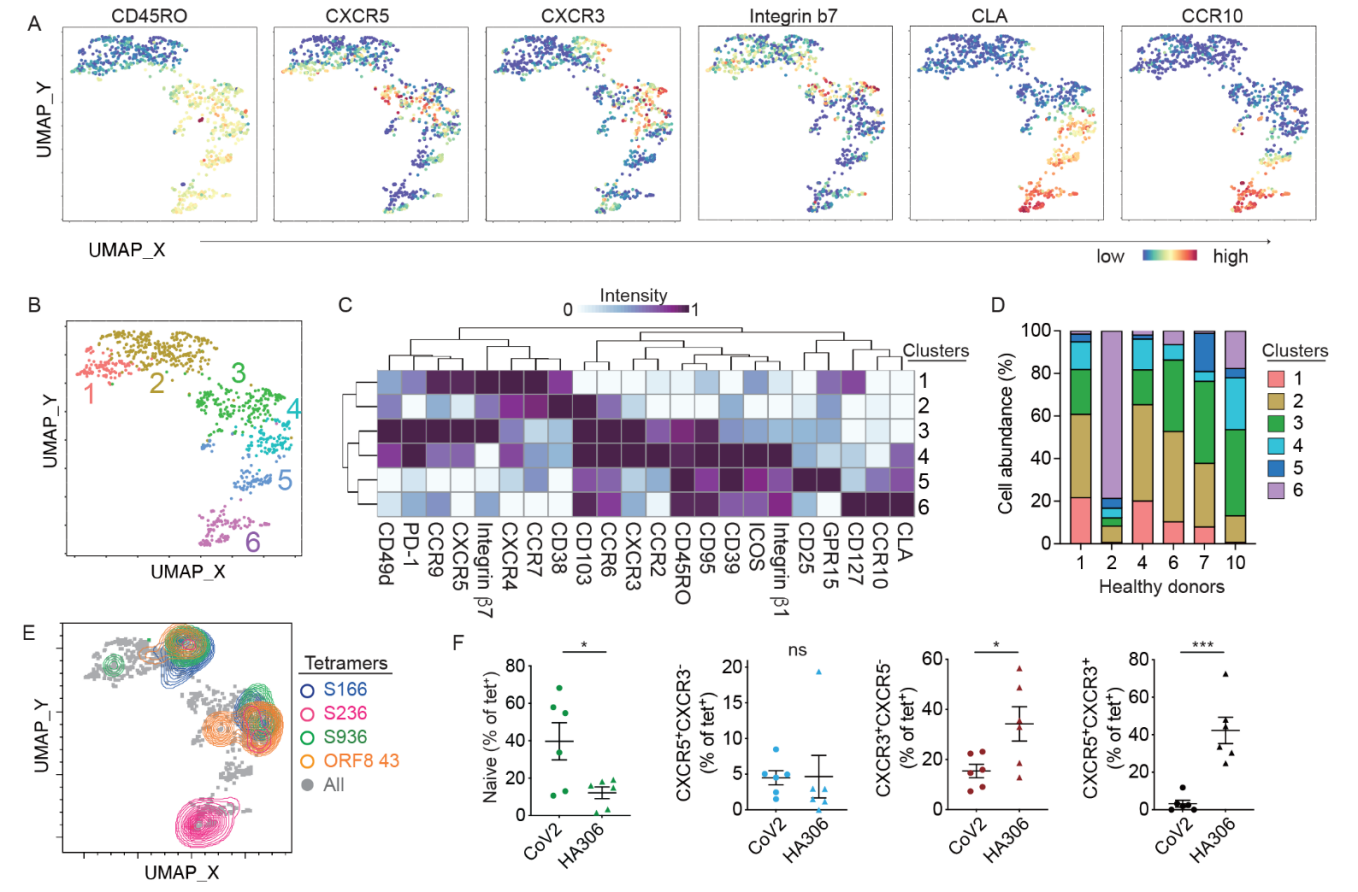
SARS-CoV-2-specific CD4^+^ T cells in unexposed donors are phenotypically heterogeneous. (A-B) UMAPs display the staining intensity of the indicated markers (A) and Phenograph defined clusters (B). Data combined 932 SARS-CoV-2-specific T cells from 6 healthy individuals that were identified using S166, S236, S936, ORF8 43 tetramers or a pool of 12 SARS-CoV-2 peptide loaded tetramers. (C) Heatmap shows the median staining signal of individual markers for clusters shown in B. (D) Bar-graph shows the relative cluster abundance within tetramer^+^ cells from each donor. (E) Individually labeled tetramer^+^ cells of the indicated specificity are projected onto an UMAP that includes all SARS-CoV-2-specific T cells. (F) Plots summarize the abundance of indicated subsets within SARS CoV-2 (CoV2) and HA306 tetramer-labeled CD4^+^ T cells. Each symbol represents averaged data from one donor based on two independent experiments (n = 6). Paired *t* tests were performed. Mean ± SEM. * p < 0.05, *** p < 0.001 and **** p < 0.0001.

To further investigate the differentiation state and phenotypic diversity of SARS-CoV-2-specific T cells at baseline, we designed a 27 fluorochrome spectral cytometry panel that focused on trafficking and chemokine receptor expression. We pooled twelve SARS-CoV-2 tetramers on the same fluorochrome to maximize the capture efficiency of SARS-CoV-2-specific T cells from a limited amount of pre-pandemic blood sample from six healthy donors. Staining with a few select tetramers was performed separately on cells from three of these individuals with more stored samples. Tetramers loaded with HA306 peptide were included for comparison. In total, 932 SARS-CoV-2 tetramer labeled T cells were identified by manual gating and combined for analyses using the Spectre pipeline (*44*). SARS-CoV-2-specific T cells expressed CD45RO, integrin β7, and CCR9 as expected (Fig. 3A, Fig. S3). A subset of tetramer^+^ cells also expressed typical markers of follicular helper (Tfh) T cells (CXCR5) or Th1 cells (CXCR3), suggesting polarization of some pre-existing memory cells into defined T helper subsets (Fig. 3A). Cutaneous lymphocyte-associated antigen (CLA) and CCR10 guide T cell trafficking to the skin (*45*–*47*). We observed high expression of these markers in some SARS-CoV-2-specific T cells (Fig. 3A), raising the possibility that relevant prior antigen experiences could extend beyond the intestinal compartment to involve other barrier tissues. Phenotypic relationship between cells were analyzed using Phenograph and visualized on UMAP (*48*, *49*). Phenograph clustered tetramer^+^ cells into six subpopulations (Fig. 3B), which were shown in a heatmap by median intensity of individual markers (Fig. 3C). SARS-CoV-2 tetramer^+^ cells were broadly divided into naïve or memory subsets by CD45RO and CCR7 staining (naïve: clusters 1 and 2, memory: clusters 3 - 6). Memory phenotype clusters were further separated by gut and skin trafficking receptors, which localized to distinct parts of the UMAP and co-stained with chemokine receptors in distinct patterns (Fig. 3A-C). Subdividing tetramer^+^ cells by donor of origin showed variable distribution of different clusters within cells from different individuals (Fig. 3D).

To investigate how different SARS-CoV-2-specific populations contributed to phenotypic heterogeneity, we examined T cells labeled individually with S166, S236, S936, or ORF8 43 tetramers. This showed that T cells stained by a particular tetramer can have a range of phenotypes and localized to distinct regions of UMAP (Fig. 3E, Fig. S4A). Between T cells stained by different tetramers, we observed both shared and unique features. For example, S166, S936, and ORF8 43 tetramer^+^ cells expressed overlapping phenotypes and were dominated by cells in clusters 2, 3, and 4 (Fig. S4B). By contrast, S236-labeled T cells differed from other SARS-CoV-2-specific populations and primarily mapped to cluster 6 on the UMAP (Fig. 3E, Fig. S4A-B). Variations between precursor populations could have reflected differences in prior antigen experiences. However, as a group, there may be similarities that distinguish them from classic memory cells generated in the context of infection and/or vaccination. To test this, we compared SARS-CoV-2 tetramer^+^ and HA306-tetramer^+^ T cells from the same individuals (Fig. 3F, Fig. S4C). Memory tetramer^+^ cells were subdivided by CD25 and CD127 expression into cells with (CD25^hi^CD127^lo^) or without (CD25^lo^) Treg features. CD25^lo^ cells were further gated by CXCR5 and CXCR3 staining to mark Tfh and Th1-associated phenotypic subsets (Fig. S4C). We did not focus on Tregs due to low frequency of CD25^hi^CD127^lo^ cells in both HA- and SARS-CoV-2 tetramer-labeled populations. For CXCR3 and CXCR5, SARS-CoV-2-specific T cells contained a lower proportion of CXCR3^+^CXCR5^+^ and CXCR3^+^CXCR5^-^ cells, but the percentage of CXCR3^-^CXCR5^+^ subset was not significantly different between SARS-CoV-2 precursors and HA-specific T cells (Fig. 3F). Collectively, these data revealed distinct features of the pre-existing memory pool. SARS-CoV-2-specific T cells expressed receptors that enable access across barrier tissues and included small subsets of Th1 and Tfh polarized cells in unexposed individuals.

### SARS-CoV-2-specific T cells responded to predicted microbial peptides

**Fig. 4.**
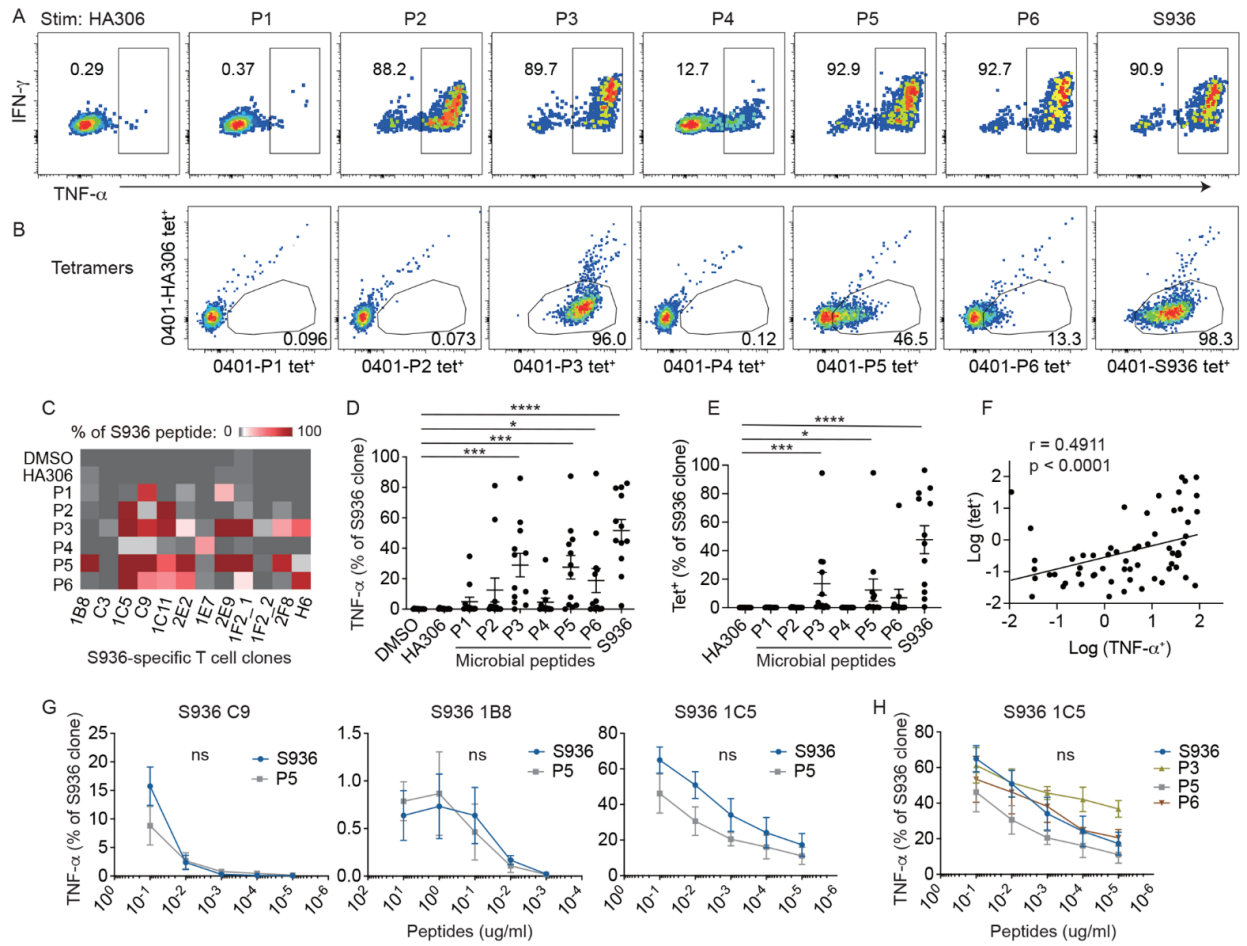
SARS-CoV-2-specific T cells respond to microbial peptides. (A) T cell response by S936 1C5 clones after a 5-hour stimulation with DCs treated with the indicated peptides. Responding T cells were identified by intracellular cytokine staining for TNF-α. (B) tetramer staining of the same clone using the indicated tetramers. Plots represent one of two independent experiments. (C) Heatmap shows T cell response to PBS or the indicated peptides as a percentage of TNF-α response to S936 for 12 S936-specific T cell clones. (D-E) Plots summarize the % of cells within a clone that responded to the indicated peptides in a stimulation assay (D) or by tetramer staining (E). Each symbol represents measurements from one clone, repeated 2-3 times. (F) The relationship between responses to P1 - P6 by TNF-α production and binding to the same peptide by tetramer staining. (G) Dose response curves of three T cell clones stimulated with decreasing concentration of S936 or P5 peptides. (H) Dose response curve of clone 1C5 stimulated with S936, P3, P5, and P6. Data are representative of 3 independent experiments. For (D), (E), and (H), Friedman test with Dunn’s multiple comparisons test was used. For (F), Spearman correlation was computed. Line represents least square regression line. For (G), Multiple Mann-Whitney test was used with Holm-Sidak’s multiple comparison procedure. Mean ± SEM. * p < 0.05, *** p < 0.001, **** p < 0.0001.

While T cells recognize antigens in a highly specific manner, TCRs can also flexibly dock onto pMHC complexes (*50*, *51*). This feature of the TCR that allows a single TCR to bind multiple distinct pMHCs is referred to as cross-reactivity. Here, we investigated whether SARS-CoV-2-specific T cells could cross-react with commensal bacteria-derived antigens using single-cell derived T cell clones. We initially focused on T cells that recognized the spike amino acid sequence 936-952 (S936) as a population that showed robust integrin β7 and CCR9 expression. We sorted single S936 tetramer^+^ cells individually into 96-well plates using pre-pandemic cells from HD1 and HD10 (Table S5). Expanded T cells were re-stained by tetramers and tested for response to S936 peptide. In total, we generated 17 clones and confirmed the correct specificity for 16 clones by tetramer staining and/or peptide response (Fig. S5). Six candidate peptides from commensal bacteria identified based on similarity to the predicted S936 core sequence were used to investigate the ability of S936-specific T cells to respond to microbe-derived sequences (Table S3). We tested twelve S936 clones that grew well in culture for TNF-α production after a 5-hours stimulation with peptide-treated monocyte-derived DCs. This revealed high levels of responses to non-spike sequences. S936 clones were most responsive to P3, P5, and P6, and had cross-reactivity to at least one microbial peptide per clone (Fig. 4A, 4C-D). A subset of these interactions probably had high avidity and could also be detected by tetramer staining (Fig. 4B). We identified several S936 clones that also bound P3 and P5 loaded tetramers and showed a positive correlation between tetramer staining and the robustness of cytokine response (Fig. 4E-F). To further quantify the responsiveness to cross-reactive peptides, we stimulated three clones with different levels of sensitivity to S936 with decreasing concentration of cognate and cross-reactive P5 peptides. Functional avidity was not significantly different between S936 and P5 sequences (Fig. 4G, Fig. S6). We further evaluated the high avidity 1C5 clone using additional bacterial sequences and found largely comparable sensitivity to cognate and cross-reactive sequences (Fig. 4H, Fig. S6). Collectively, these data demonstrated that SARS-CoV-2-specific T cells can recognize and respond to non-viral sequences. Cross-reactivity involved a substantial number of virus-specific precursors and included cells with high avidity to SARS-CoV-2.

### SARS-CoV-2-specific T cells responded to fecal and bacterial lysates

**Fig. 5.**
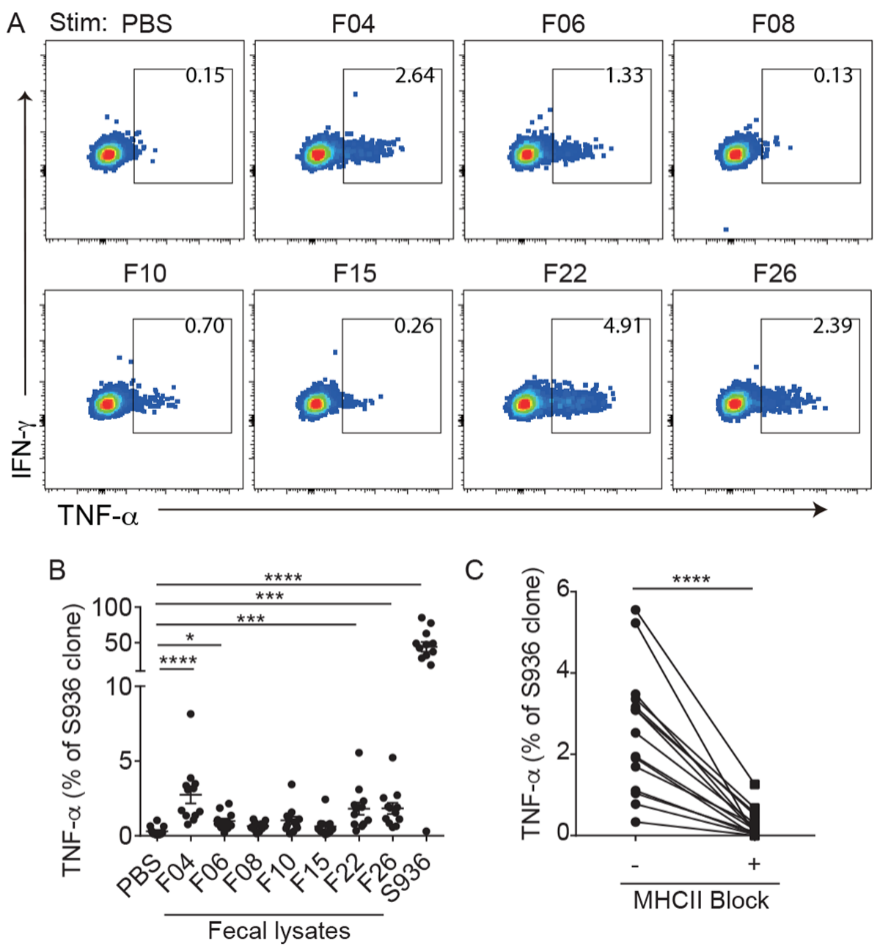
SARS-CoV-2-specific T cells respond to fecal lysates. (A) Plots show TNF-α response by the S936 2F8 clone after a 5-hour stimulation with DCs treated with PBS, fecal lysates from healthy adults (F04, F06, F08, F10, F15, F22, F26), or the cognate peptide. (B) Plots summarize the % of cells within a clone that responded to the indicated treatment. Each symbol represents measurements from one clone (n = 12), repeated 2-5 times. (C) The frequency of TNF-α^+^ produced by 5 clones in response to F04, F22, and F26 with or without anti-MHC class II blocking antibodies. Data are representative of 3-5 independent experiments. (B) Friedman test with Dunn’s multiple comparisons test was used. (C) Paired *t* test was used. Mean ± SEM. * p < 0.05, *** p < 0.001, **** p < 0.0001.

**Fig. 6.**
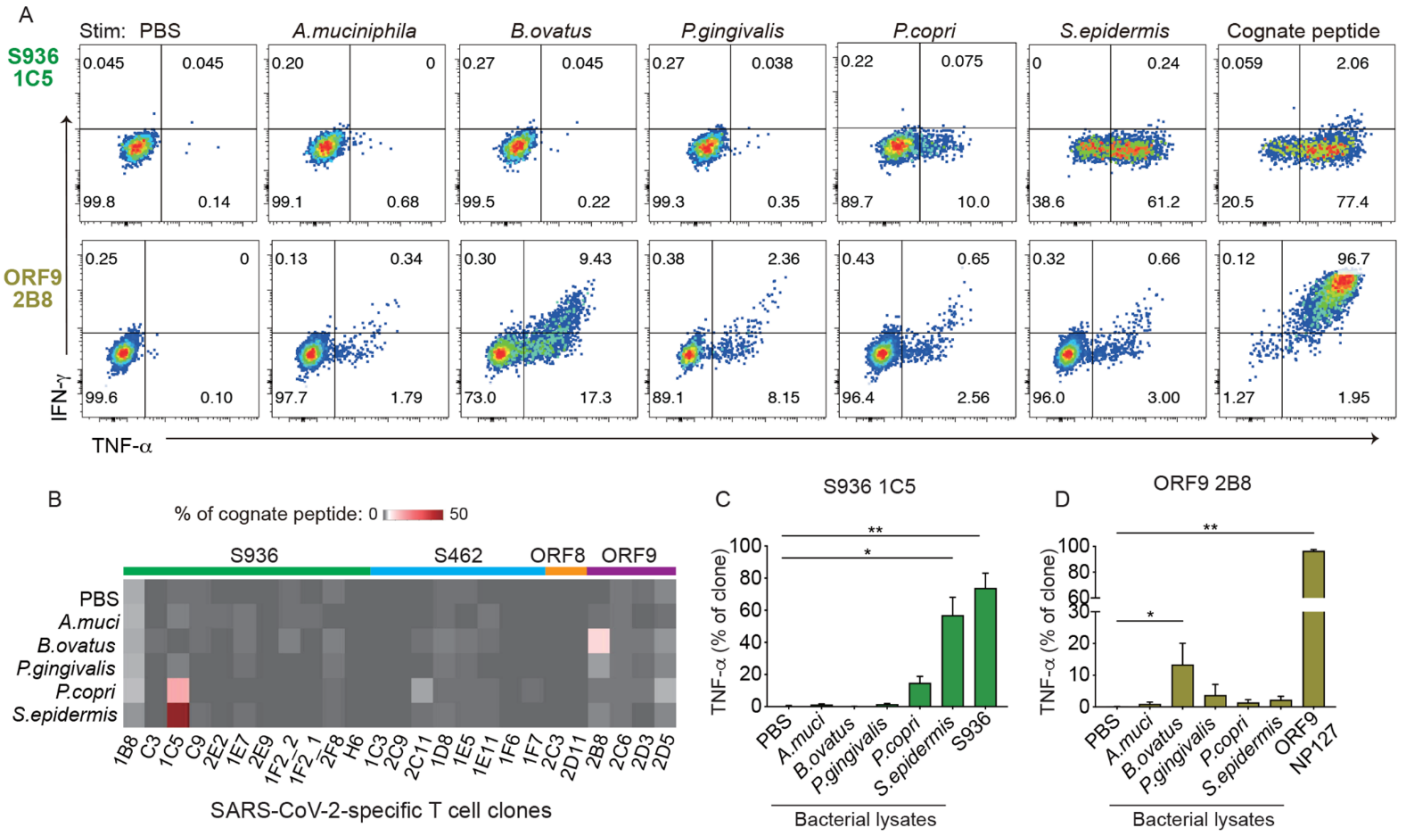
SARS-CoV-2-specific T cells respond to lysates from gut and skin bacteria. (A) Cytokine response after stimulation by PBS or the indicated bacterial lysates. Plots represent one of four (S926 1C5) or three (ORF9 2B8) independent experiments. (B) Heatmap shows T cell response to PBS or the indicated lysates as a percentage of TNF-α response to the cognate peptide, repeated 2-4 times. SARS-CoV-2 specificity is indicated above the heatmap and separated by color. (C-D) Bar graphs show the % of cells within 1C5 (C) or 2B8 (D) that produced TNF-α in response to PBS, bacterial lysates, or the cognate peptide. Friedman test with Dunn’s multiple comparisons test was used for (C) and (D). Mean ± SEM. * p <0.05 ** p <0.01.

Next, we examined cross-reactivity to naturally occurring microbial components. S936 T cell clones were stimulated with DCs incubated with fecal lysates from 7 healthy individuals. This showed a significant response to 4 fecal lysates (F04, F06, F22, F26) compared to phosphate buffered saline (PBS) (Fig. 5A-B). Pre-treating lysate loaded DCs with major histocompatibility complex II (MHC II) blocking antibodies inhibited TNF-α production, indicating that cytokine response to these fecal lysates was MHC-dependent (Fig. 5C, Fig. S7). While these data provided evidence that a SARS-CoV-2-specific T cell clone could recognize naturally processed microbial epitopes, the relevant sources of antigens were challenging to identify due to the complexity of microbes in the stools. To analyze more defined bacterial antigens, we cultured commensal bacteria and generated lysates for stimulation. We broadened our analyses to include bacteria from different barrier tissues, using *Porphyromonas gingivalis* from the oral cavity, *Prevotella copri, Bacteroides ovatus, and Akkermansia muciniphilia* from the gut, and *Staphylococcus epidermidis* from the skin. To determine if SARS-CoV-2-specific T cells besides those recognizing S936 could respond to microbial antigens, we generated additional T cell clones for other SARS-CoV-2 epitopes and confirmed the specificity of tetramer-stained cells as before. Single S462, ORF8 43, or ORF9 NF127 tetramer^+^ cells were sorted and expanded in culture. Antigen-specificity was validated for 94% of the clones by staining with the same tetramer and/or response to the cognate peptide (9/9 S462 clones, 2/2 ORF8 clones, 4/5 ORF9 clones, Fig. S8). In total, 25 SARS-CoV-2-specific T cell clones were stimulated with five cultured bacterial lysates. This identified two responsive clones from different donors that recognized distinct SARS-CoV-2 epitopes (Fig. 6A-B). The S936-specific 1C5 clone from HD10 responded robustly to *S. epidermidis* and moderately to *P. copri* (Fig. 6C). The 2B8 clone from HD5 recognized ORF9 NF127 and showed preferential cross-reactivity to *B. ovatus* (Fig. 6D). These data provided evidence that SARS-CoV-2-specific T cells from unexposed individuals could cross-react with naturally processed antigens from commensal bacteria on the skin and in the gastrointestinal tract.

### Ex vivo detection of cross-reactive T cells

**Fig. 7.**
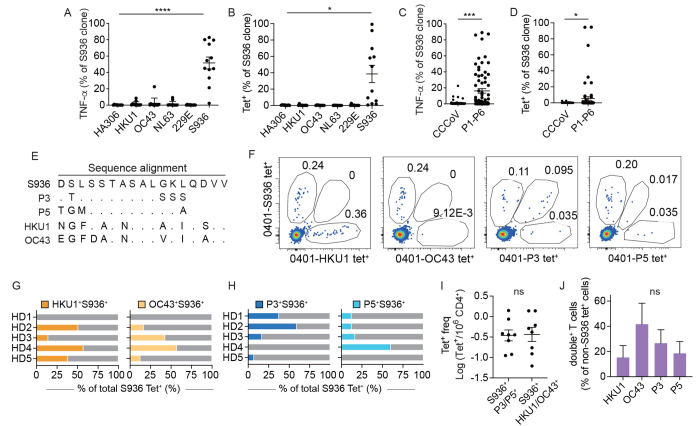
T cell cross-reactivity to CCCoV. (A-B) The % of cells within a clone that responded to homologous sequences from CCCoV by TNF-α production (A) or tetramer staining (B). Each symbol represents measurements from one clone (n = 12), repeated 2-3 times (A) or 1-2 times (B). (C-D) Plots summarize the frequency of response to a CCCoV peptide (HKU1, OC43, NL63, or 229E) or a bacterial peptide (P1-P6) by TNF-α response (C) or tetramer staining (D). (E) Sequence alignment of S936 from SARS-CoV-2 with bacterial sequences P3, P5, and homologous CCCoV sequences from HKU1 and OC43. (F) Identification of CD4^+^ T cells from HD1 that recognized S936, HKU1, OC43, P3, or P5 peptides by direct ex vivo tetramer staining. Plots represent one of two independent experiments. (G) Bar-graphs show the frequency of T cells that cross-reacted with HKU1 or OC43 sequences as a percentage of total S936 tetramer^+^ cells. (H) Cross-reactivity to P3 and P5 as a percentage of total S936 tetramer^+^ cells. Each bar represents data from one individual. (I) The frequency of S936-specific T cells that cross-reacted with bacterial sequences (P3 or P5) or CCCoV peptide (HKU1 or OC43). (J) S936^+^HKU1^+^, S936^+^OC43^+^, S936^+^P3^+^, or S936^+^P5^+^ cells as a percentage of total HKU1, OC43, P3, or P5 tetramer^+^ T cells. For G-J, data were generated from 1-2 independent experiments depending on cell availability. (A-B) Friedman test with Dunn’s multiple comparisons test was used. (C-D) Mann-Whitney test was used. (I) Welch’s *t* test was used. (J) Kruskai-Wallis test and Dunn’s multiple comparisons test were used. Mean ± SEM. * p < 0.05, *** p < 0.001. **** P < 0.0001.

CCCoV is a known source of cross-reactive antigen that contributes to pre-existing responses to SARS-CoV-2 (*24*) (*16*, *25*). Here, we compared cross-reactivities to bacterial peptides with responses to CCCoV to gain insights into the relative contribution of different types of exposures. S936 T cell clones were stimulated with homologous sequences from HKU1, OC43, NL63, and 229E. To our surprise, DCs treated with CCCoV peptides activated fewer T cell clones compared to bacterial sequences (Fig. 7A, 7C). None of the S936 T cell clones analyzed showed a positive staining for the CCCoV tetramers (Fig. 7B, 7D). To eliminate the possibility that the lack of CCCoV response was due to poor growth of this type of cross-reactive cells in culture, we performed direct ex vivo tetramer staining on cells that were used to generate the clones from HD1. We produced tetramers for two bacterial and two CCCoV peptides (Fig. 7E). The beta coronaviral sequences from HKU1 and OC43 were used because they were most similar to S936. Bacterial peptides P3 and P5 were selected because they stimulated greater numbers of T cell clones. In agreement with T cell clones generated from the same donor, we found S936 tetramer^+^ T cells that bound P3 and P5 tetramers but not HKU1 or OC43 tetramers by direct ex vivo staining (Fig. 7F, S8A). To test if the lack of CCCoV cross-reactivity was donor-specific, we expanded our analyses to include cells from HD2, HD3, HD4, and HD5. Tetramer staining identified S936^+^CCCoV^+^ double tetramer positive cells in these donors (Fig. 7G, S9A). The type and degree of cross-reactivity varied between individuals but showed a similar overall frequency between bacterial and CCCoV sequences (Fig. 7G-I). As a percentage of HKU1, OC43, P3, or P5 tetramer^+^ populations, S936 cross-reactivity was not significantly different between CCCoV or bacterial peptide-specific T cells (Fig. 7J, Fig. S9B). These data provided further evidence in support of cross-reactivity between SARS-CoV-2 and bacterial antigens. Interactions with commensal bacteria could be a major source of antigen-experience that complements CCCoV in the development of pre-existing memory to SARS-CoV-2.

## DISCUSSION

T cells that recognize SARS-CoV-2 are found in unexposed individuals (*15*–*22*), but the composition of these cells remain poorly defined. Here, we examined the pre-existing state of 117 SARS-CoV-2-specific CD4^+^ T cell populations in healthy adults. A tetramer-based enrichment approach was used to analyze rare antigen-specific T cells directly ex vivo. This approach enabled quantitative analyses of T cell frequency and cellular states with minimal perturbation. The specificity of tetramer staining was confirmed for over 90% of labeled cells using single cell-derived clones generated from individually sorted tetramer^+^ cells. The experiments were performed using blood collected before year 2020 to ensure the absence of prior SARS-CoV-2 exposure. Our analyses of SARS-CoV-2-specific T cells showed variation in precursor frequencies across donors and between T cells that recognized the same antigen. Among SARS-CoV-2 epitopes surveyed, T cell frequency was highest for a peptide from ORF8, an immune modulatory protein that shared little sequence homology with CCCoV (*52*). Irrespective of the degree of similarity to endemic coronaviruses, we were able to identify memory phenotype T cells that recognized SARS-CoV-2 epitopes. Overall, over 60% of SARS-CoV-2-specific T cells examined acquired a memory phenotype in unexposed individuals.

While prior exposures to endemic coronaviruses influence the pre-existing repertoire of SARS-CoV-2-specific T cells, the high proportion of pre-existing memory T cells remains incompletely explained. Because a TCR can interact with peptide-MHC ligand with considerable flexibility, we sought to investigate cross-reactivity to other environmental antigens beyond related viruses. The intestinal tract is home to trillions of microbial organisms that influence health and disease (*13*, *39*, *40*, *53*). Similarly, the skin houses a highly diverse microbial community with essential immune functions (*54*). Using trafficking receptor expression to infer tissue tropism, we performed phenotypic analyses of ex vivo isolated SARS-CoV-2-specific CD4^+^ T cells and identified skin and gut-tropic populations. Whether SARS-CoV-2-specific T cells could cross-react with microbial antigens in these tissue compartments were evaluated using T cells clones and by tetramer staining. These studies revealed specific T cell recognition and response to microbial peptides, stool lysates, and defined bacteria from skin and gastrointestinal tract.

Intriguingly, a substantial portion of SARS-CoV-2-specific CD4^+^ precursor cells could recognize microbial antigens. A subset of these cross-reactive interactions had high functional avidity and bound bacterial epitopes with sufficient strength to be detected by tetramers. Cross-reactivity to microbial peptides were heterogeneous but were also readily detectable as a group and showed a similar overall frequency as homologous CCCoV sequences in our experimental system. As our data were generated from limited sampling of SARS-CoV-2-specific populations and small numbers of T cell clones, they provided a selective view of the pre-existing repertoire which likely contains other T cells with different cross-reactive potentials. Another limitation is that we do not know if SARS-CoV-2-specific T cells had in fact encountered a particular bacterial antigen and the timing or the order of these exposures. Relevant antigens could also come from fungal and viral components of the microbial environment that were not analyzed in this study. Broader studies that link microbiome composition with antibody and T cell responses in people of different age groups living in distinct environments would further inform the development of human pre-existing repertoire to novel pathogens.

How pre-existing states influence host protection remain incompletely understood. The presence of a pre-existing pool of T cells that can recognize SARS-CoV-2 has generated a great deal of excitement for the possibility that they might provide superior responses to infections (*15*–*22*). The frequency of pre-existing SARS-CoV-2-specific T cells are associated with beneficial features such as milder disease and abortive infection (*55*–*57*). However, SARS-CoV-2 T cells from unexposed individuals required more peptides to respond in culture, a feature that was shared with T cells from patients with severe COVID-19 infection (*22*). By analyzing the phenotype of tetramer-labeled T cells, we showed that pre-existing memory pool contained a diverse population of SARS-CoV-2-specific T cells with distinct differentiation features and trafficking potentials. The heterogeneity within the pre-existing repertoire is consistent with priming by various cross-reactive sources and may contribute to the diversity of responses after exposure. We speculate that the expression of trafficking receptors may enable accelerated migration of pre-conditioned memory T cells into tissues to enhance local defense in the event of infection. Pre-existing Tfh cells might orchestrate B cells to further jump start responses to a pathogen. Alternatively, hyperinflammatory cells that infiltrate tissues may contribute to acute and/or persistent damage. Extension of T cell cross-reactivity to self-antigens may perpetuate immune processes and result in maladaptive host responses after viral clearance. Cross-reactive T cells activated by COVID-19 infection may in turn target the microbiome and further modify the relationship between microbes and the immune system. Future studies will be needed to investigate how pre-existing populations respond to perturbations and converge to modulate the overall quality of immune response to pathogens.

In summary, our analyses of SARS-CoV-2 precursor repertoire showed that pre-existing SARS-CoV-2 specific memory T cells express diverse phenotypes and display broad tissue tropism. By examining the cross-reactivities of individual T cells, our data further revealed a wide range of responses to commensal bacteria. These findings highlight the breadth of T cell recognition and suggest that life-long exposures to a diverse microbial environment could have profound impacts on the composition of the immune repertoire. Pre-existing immune memory is typically studied in the context of exposure to related pathogens. Our data provide the basis for considering a broader range of antigen-experiences in the education of the baseline immune responses. Inter-individual differences in the abundance and differentiation states of pre-existing cells resulting from different life experiences may contribute to the heterogeneity of human responses to SARS-CoV-2 and potentially other infections.

## MATERIALS AND METHODS

### Study Design

The goal of the study was to define the pre-existing state of SARS-CoV-2-specific T cells in unexposed individuals. Cryopreserved cells were stored from past collection of de-identified donors from the Stanford Blood Bank or from prior studies at the University of Pennsylvania (*30*). Subject characteristics are shown in Table S1. Stool samples were baseline samples from a controlled feeding study in healthy adult volunteers (aged 18-60 years) (*58*). All samples were de-identified and obtained with IRB regulatory approval from the University of Pennsylvania.

### Direct ex vivo T cell analyses and cell sorting

HIS-tagged HLA-DR protein monomers with tethered thrombin cleavable CLIP peptide were produced by Hi5 insect cells and extracted from culture supernatant using Ni-NTA (Qiagen). HLA-DR monomers were biotinylated overnight at 4°C using BirA (Avidity), analyzed by streptavidin gel-shift assay to confirm biotinylation, and purified by size exclusion chromatography using Superdex 200 size exclusion column (AKTA, GE Healthcare). Peptide exchange and tetramerization were performed using standard protocols as previously described (*28*, *59*). In brief, HLA-DR proteins were incubated with thrombin (Millipore) at room temperature for 3 - 4 hours and exchanged with peptides of interest in 50-fold excess at 37°C for 16 hours. Peptide loaded HLA-DR monomers were incubated with fluorochrome-conjugated streptavidin at 4 - 5: 1 ratio for 2 min at room temperature, followed by a 15 min incubation with an equal volume of biotin-agarose slurry (Millipore). Tetramers were buffered exchanged into PBS, concentrated using Amicon ULTRA 0.5ml 100KDa (Millipore), and kept at 4°C for no more than 2 weeks prior to use. For tetramer staining, 30 to 90 million CD3 or CD4 enriched T cells were stained at room temperature for 1 hour using 5 ug of each tetramer in 50 to 100 ul reaction. Tetramer tagged cells were enriched by adding anti-fluorochrome and anti-HIS MicroBeads (MiltenyiBiotec) and passing the mixture through LS columns (MiltenyiBiotec). Column-bound cells were washed and eluted according to manufacturer protocol. For antibody staining, eluted cells were stained with viability dye (ThermoFisher) and anti-CD19 and anti-CD11b (BioLegend) to exclude dead and non-T cells. Other antibodies used are listed in Table S6. Surface staining was carried out in 50 to 100ul of FACS buffer (PBS plus 2% FCS, 2.5mM EDTA, 0.025% Sodium Azide) for 30 min at 4°C. Samples were acquired by flow cytometry using LSRII (BD) or sorted on FACS Aria (BD). Frequency calculation was obtained by mixing 1/10^th^ of pre- and post-enrichment samples with 200,000 fluorescent beads (Spherotech) for normalization (*28*). Non-zero populations were included in the analyses performed using FlowJo (BD). Spectral flow cytometric analyses were performed with following modifications: For pooled tetramer staining, 2ug of tetramers loaded with each of the twelve SARS-CoV-2 peptides used in this study were tagged to the same fluorochrome and combined in the staining reaction. Tetramer enriched cells were stained with live/dead dyes, exclusion markers, and a panel of additional surface antibodies (Table S6) for 1h at 4°C followed by fixation with 2% paraformaldehyde. Samples were acquired on spectral flow Cytex AURORA (ARC 1207i).

### Microbial culturing and preparation of lysates

*Prevotella copri* DSMZ 18205, *Staphylococcus epidermidis* ATCC 14990, *Akkermansia muciniphila* ATCC BAA-835, *Porphyromonas gingivalis* ATCC 33278, and *Bacteroides ovatus* ATCC 8483 were obtained from DSMZ (Braunschweig, Germany) or ATCC (Manassas, VA) and grown in Schaedler Broth, Nutrient Broth, Brain Heart Infusion Broth, Supplemented Tryptic Soy Broth, or Modified Chopped Meat Medium, respectively. *P. copri, A. muciniphila*, and *B. ovatus* were grown in an anaerobic glove box containing approximately 2.5% CO_2_, 2.5% H_2_, and 95% N_2_. All strains were grown at 37°C until the mid- to late-log phase growth. 250 ml of the culture was spun down at 3000 g for 10 min, the supernatant was removed, the pellet was resuspended in 4 ml of PBS, and sonicated using a probe sonicator (30 s followed by 1 min rest, repeated 3 times) for the preparation of bacterial lysates. The bacterial lysates were clarified by centrifugation at 10,000 rpm for 1 min at 4°C. The clarified bacterial lysates were then treated with Polymyxin beads (Sigma) to remove endotoxin content followed by estimation of total protein content in the lysates through BCA protein assay method (ThermoFisher). Fecal lysates were generated by diluting 100 mg of fecal sample in 1 ml PBS and sonicated 3 times for 1 min at 4°C with 30 s of rest in between. The fecal lysates were clarified by centrifugation at 15000 rpm for 5 min before use.

### Generation and stimulation of T cell clones

Generation of T cell clones: Cells were stained with tetramers and enriched by magnetic isolation as described above. Tetramer-positive cells were sorted as single cells into 96-well plates using the purity mode on FACSAria II (BD). Each well contained 10^5^ irradiated PBMCs and 10^4^ JY cell line (an Epstein–Barr virus (EBV)-immortalized B cell lymphoblastoid line, ThermoFisher), PHA (1:100, ThermoFisher), IL-7 (25 ng/ml, PeproTech), and IL-15 (25 ng/ml, PeproTech). IL-2 (50 IU/ml, PeproTech) was added on day 5 and replenished every 3-5 days. Cells were resupplied with fresh medium with IL-2 (50 IU/ml), PHA (1:100) and 10^5^ irradiated PBMCs every two weeks.

Generation of DCs: Monocytes from PBMCs of HD1 and HD2 were isolated using RosetteSep Human Monocyte Enrichment Cocktail (StemCell). 5 million cryopreserved monocytes were thawed in 6.4 ml of DCs media (RPMI 1640 plus Glutamine,10% FCS, Pen/Strep, HEPES) containing 100 ng/ml GM-CSF and 500 U/ml IL-4. On the third day, 3.2 ml fresh media with 100 ng/ml GM-CSF, 500 U/ml IL-4, and 0.05 mM 2-mercaptoethanol were added to the ongoing culture. For maturation of DCs with bacterial lysates or fecal lysates, the immature DCs were harvested on day 4 after thawing. 25,000 DCs were plated in U bottom 96-well plate, incubated with 50 ug/ml polymyxin bead treated bacterial lysates or 50 ug/ml fecal lysates for 36h, followed by the addition of LPS (100 ng/ml) on day 6. The bacterial lysates or fecal lysates treated DCs were co-cultured with T cell clones on day 7. For peptide stimulation, immature DCs were harvested on day 5 after thawing. 0.1 million DCs were plated in a flat bottom 96-well plate and matured with LPS (100 ng/ml) and incubated with peptide (20 ug/ml) for 16-24h before co-culturing with T cells.

Stimulation of T cell clones: T cell clones were rested overnight in fresh media without IL-2 and added to wells containing matured DCs. Cells were incubated for 5 hours in the presence of monensin (2 uM, Sigma) and Brefeldin A (5 ug/ml, Sigma). For MHC blocking experiments, anti-MHC class II antibodies, L243 (0.37 ug/ul, BioLegend) and TU39 (0.01 ug/ul, BioLegend), were added to T cells before combining with DCs. Intracellular cytokine staining with anti-TNF-α and anti-IFN-γ (BioLegend) was performed after stimulation using BD Cytofix/Cytoperm Fixation/Permeabilization Kit according to manufacturer protocol (BD).

### Bacterial and CCCoV sequence analyses

Bacterial peptides: To identify potential cross-reactivity to microbial peptides, we predicted the DR4 binding register in S936 using NetMHCII 2.3. We then performed a Protein BLAST analyses against the non-redundant protein sequence database using the S936 core sequence (LSSTASALG), excluding SARS-CoV-2 and coronavirus related sequences (taxid: 2901879, 2697049, 694009, 2697049, 1508227, 694002, 694003, 1928434). We curated six sequences from commensal bacteria allowing for 1 amino acid mismatch and extended these 9-mer peptides by two amino acids on both ends based on the bacterial protein sequence.

Coronavirus sequences: All spike and non-spike sequence of SARS-CoV-2 and common coronaviruses were derived from NCBI database. (SARS-CoV-2 spike: YP_009724390.1; SARS-CoV-2 ORF8: YP_009724396.1; SARS-CoV-2 NP: QSM17284.1; HKU1 spike: ABD96198.1; HKU1 ORF8: AZS52623.1; HKU1 NP: YP_173242.1; 229E spike: AWH62679.1; 229E NP: P15130.2; NL63 spike: APF29063.1; NL63 NP: Q6Q1R8.1; OC43 spike: QEG03803.1; OC43 NP: P33469.1). Pair-wise alignment using Clustal Omega was performed to identify aligned regions. Similarity between the aligned sequences were calculated by Sequence Identity And Similarity (SIAS) using BLOSUM62 matrix. A score of 0 was used if no aligned region was found. Missing sequences were excluded from the analyses.

### High-dimensional phenotypic analyses

Spectral cytometric data were analyzed by manual gating to select SARS-CoV-2 tetramer-labeled cells from each sample. Tetramer^+^ cells stained using pooled or individual tetramers were exported from Flowjo. A total of 932 tetramer^+^ cells were read into R by flowCore and combined into one single dataset for subsequent data processing and high-dimensional analyses using the Spectre package in R (*42*). Staining intensities were converted using Arcsinh transformation with a cofactor of 2000. Batch alignment was performed by first coarse aligning the batches with quantile conversions of marker intensities calculated with a reference sample included in all the batches and then applying this conversion to all samples via the CytoNorm algorithm. Clustering was performed using Phenograph with nearest neighbors set to 60 (k = 60) (*44*). Unbiased uniform manifold approximation and projection (UMAP) was used for dimensional reduction and visualization (*43*). Color scale was modified to use the same color for 0 and values under 0 after Arcsinh transformation.

### Statistical Methods

Data transformation was performed using the logarithmic function or inverse hyperbolic sine transformation ( $\sinh^{-1}\left(X\right)=\ln\left(X+\sqrt{\left(1+X^{2}\right)}\right)$ ) when data contained zero values. Assessment of normality was performed using D’Agostino-Pearson test. Spearman correlation was used if either of the two variables was non-normal. Otherwise, Pearson correlation was used to measure the degree of association. The best-fitting line was calculated using least squares linear regression. Statistical comparisons of two means were performed using two-tailed Student’s *t* test or paired *t* test using a p-value of < 0.05 as the significance level. P-values were adjusted for multiple comparisons. Multiple comparisons procedures were performed when the Welch’s one-way ANOVA (Dunnett’s T3), Friedman’s repeated measures one-way ANOVA (Dunn), or two-way ANOVA (Tukey) was significant. Statistical analyses were performed using GraphPad Prism. Lines and bars represent mean and variability is represented by standard error of the mean (SEM). * P < 0.05, ** P < 0.01, *** P < 0.001, **** P < 0.0001, ns (not significant).
