# Supplementary material for: SARS-CoV-2-specific T cells in unexposed adults display broad trafficking potential and cross-react with commensal antigens
Source: Sci Immunol. 2022 Jul 14:eabn3127. doi: 10.1126/sciimmunol.abn3127 (PMC9348748; doi:10.1126/sciimmunol.abn3127)
Supplement: Supplementary file 1 — Figs. S1 to S9 Tables S1 to S6 [file sciimmunol.abn3127_sm.pdf]

Science Immunology
AAAS

Supplementary Materials for

SARS-CoV-2-specific T cells in unexposed adults display broad trafficking potential and cross-react with commensal antigens

Laurent Bartolo *et al.*

Corresponding author: Laura F. Su, Laurasu@upenn.edu

DOI: 10.1126/sciimmunol.abn3127

The PDF file includes:

Figs. S1 to S9
Tables S1 to S6

Other Supplementary Material for this manuscript includes the following:

Table S7

Title: SARS-CoV-2-specific T cells in unexposed adults display broad trafficking potential and cross-react with commensal antigens

Authors:

Laurent Bartolo[1†], Sumbul Afroz[1†], Yi-Gen Pan[1†], Ruozhang Xu[1, 2†], Lea Williams[1,2], Chin-Fang Lin[1], Ceylan Tanes[5], Kyle Bittinger[5], Elliot S. Friedman[3], Phyllis A. Gimotty[4], Gary D. Wu[3], Laura F. Su[1,2*]

Affiliations:

[1] Department of Medicine, Division of Rheumatology, Perelman School of Medicine, Institute for Immunology, University of Pennsylvania, Philadelphia, PA 19104, USA.
[2] Corporal Michael J Crescenz VA Medical Center, Philadelphia, PA, 19104, USA.
[3] Division of Gastroenterology and Hepatology, Perelman School of Medicine, University of Pennsylvania, Philadelphia, PA 19104, USA.
[4] Department of Biostatistics, Epidemiology, and Informatics, Perelman School of Medicine, University of Pennsylvania, Philadelphia PA 19104, USA.
[5] Division of Gastroenterology, Hepatology and Nutrition, Children's Hospital of Philadelphia, PA, 19104, USA.

[†] indicates co-first authors
* Corresponding author and lead contact:
Laura F. Su
University of Pennsylvania
421 Curie Blvd BRBII/III 311
Philadelphia, PA 19104
215-898-4181
215-573-6804 (Fax)
Laurasu@upenn.edu

Supplementary Materials

Figure S1: CD4⁺ T cells from unexposed individuals recognized distinct SARS-CoV-2 epitopes
Figure S2: Influenza and YFV-specific CD4⁺ T cells
Figure S3: High-dimensional phenotypic analyses of pre-immune SARS-CoV-2-specific T cells
Figure S4: Phenotypic analyses of individual SARS-CoV-2-specific populations
Figure S5: Confirmation of S936-specific T cell clones generated from single tetramer⁺ T cells
Figure S6: Functional avidity of S936-specific T cell clones in response to cognate and cross-reactive peptides
Figure S7: T cell response to fecal lysates with or without MHC inhibition
Figure S8: Confirmation of S462, ORF8, and ORF9-specific T cell clones generated from single tetramer⁺ T cells
Figure S9: Gating strategy for identifying cross-reactive T cells
Table S1: Participant information
Table S2: SARS-CoV-2 peptide sequences
Table S3: Non-SARS-CoV-2 peptide sequences
Table S4: Sequence alignment between SARS-CoV-2 and common circulating coronaviruses
Table S5: Summary of T cell clones
Table S6: Surface antibody staining panels
Table S7: Summary tables of raw data

Supplementary Material

Table S1: Participant information

Participant ID	Sex	Sample collection year	Age at the time of collection
HD1	M	2017	42
HD2	F	2019	52
HD3	M	2017	21
HD4	F	2017	22
HD5	M	2017	25
HD6	M	2011	77
HD7	F	2011	62
HD8	F	2011	61
HD9	M	2011	60
HD10	M	2011	37
HD11	M	2011	70
HD12	M	2011	32

Table S2: SARS-CoV-2 peptide sequences

Peptide	Antigen	Sequence	Percentile Rank	Reference
S116	SPIKE	SLLIVNNATNVVIKV	4.30	(*1*)
S166	SPIKE	CTFEYVSQPFLMDLE	24.00	(*1, 2*)
S236	SPIKE	TRFQTLLALHRSYLTPGDSSS	1.20	-
S326	SPIKE	IVRFPNITNLCPFGE	8.50	(*1*)
S336	SPIKE	CPFGEVFNATRFASV	18.00	(*1*)
S451	SPIKE	YLYRLFRKSNLKPFERDI	17.00	-
S462	SPIKE	KPFERDISTEIYQ	6.30	-
S936	SPIKE	DSLSSTASALGKLQDVV	12.00	-
S1011	SPIKE	QLIRAAEIRASANLAATK	2.70	-
S1151	SPIKE	ELDKYFKNHTSPDVD	11.00	(*1*)
ORF8 43	ORF8	SKWYIRVGARKSAPL	15.00	(*3*)
ORF9 NP 127	Nucleocapsid phosphoprotein	KDGIIWVATEGALNT	13.00	(*3*)

MHC II binding prediction tools from IEDB was applied, using default settings for IEDB recommended 2.2. As is option was used for peptides with less than 15 amino acids.

Table S3: Non-SARS-CoV-2 peptide sequences

Name	Sequence	Source
P1	INLSNTASALGYG	bacterioidcarboxypeptidase-like regulatory domain-containing protein [Bacteroides ovatus]/TonB-dependent receptor [Bacteroides thetaiotaomicron]
P2	VSLSSTATALGGN	hypothetical protein EOP83_02905, partial [Verrucomicrobiaceae bacterium]
P3	DTLSSTASALSSS	Ig-like domain Enterobacter sp. DE0047
P4	EELSATASALGAL	hypothetical protein DIU60_00360 [Actinobacteria bacterium]
P5	TGMSSTASALGKA	hypothetical protein EPN97_09910 [Alphaproteobacteria bacterium]
P6	SNLSSTASALSAT	MULTISPECIES: YhgE/Pip domain-containing protein [Corynebacterium]
HA306	PKYVKQNTLKLAT	Hemagglutinin protein of Influenza virus, 306-318
YF23	ESATILMTATPPGTS	Non-structure protein 3 of yellow fever virus, 313-327
YF25	KGPLRISASSAAQRR	Non-structure protein 3 of yellow fever virus, 448-462
YF42	ACLSKAYANMWSLMY	Non-structure protein 5 of yellow fever virus, 756-770
YF50	TIRVLALGNQEGSLKTA	Envelope protein of yellow fever virus, 241-257
YF51	TDKMFFVKNPTDTGHGT	Envelope protein of yellow fever virus, 301-317
YF53	GLYGNGILVGDNSFVSA	Non-structure protein 3 of yellow fever virus, 151-167

YF54	LPSIRAANVMAASLRKA	Non-structure protein 3 of yellow fever virus, 367-383
YF57	TLGEVWKRELNLLDKRQ	Non-structure protein 5 of yellow fever virus, 7-23
YF69	WILADKRPTAWFLPSIR	Non-structure protein 3 of yellow fever virus, 355-371
YF80	TGHGTVVMQVKVSKGAP	Envelope protein of yellow fever virus, 313-329
YF108	VIKILTYPWDRIEEVTR	Non-structure protein 5 of yellow fever virus, 325-341

Table S4: Sequence alignment between SARS-CoV-2 and common circulating coronaviruses

S116

				Score
SARS-CoV-2	116	S L L I V N N A T N V V I K V	130	
HKU1	136	T I V . G S V F I . N S Y T I	150	40.0
OC43	155	G V N K L Q G L L E . S V C Q	169	33.3

S462

				Score
SARS-CoV-2	462	K P F E R D I S T E I Y Q	474	
HKU1	474	C . C A K P S F A S S C K	486	15.4
NL63	450	C . . S F S K L N N F Q K	462	15.4
OC43	473	. . Q P A G V F . N H D V	485	30.8

S166

				Score
SARS-CoV-2	166	C T F E Y V S Q P F L M D L E	180	
HKU1	183	E S W H F D K S E P . C L F K	197	26.7
229E	115	D V I R . N I N F E E N L R R	129	13.3
NL63	175	. Y . N . S C V F S V V N A T	189	26.7
OC43	201	. L Y K R N F T Y D V N A T Y	215	20.0

S936

				Score
SARS-CoV-2	936	D S L S S T A S A L G K L Q D V V	952	
HKU1	1021	N G F . A . N . . . A . I . S . .	1037	58.8
229E	805	. A F T G V N D . I T Q T S Q A L	821	29.4
NL63	988	A . F . . V N D . I T Q T A E A I	1004	41.2
OC43	1029	E G F D A . N . . . V . I . A . .	1045	58.8

S236

				Score
SARS-CoV-2	236	T R F Q T L L A L H R S Y L T P G D S S S	256	
HKU1	233	S H Y Y V . P L T C N A I S S N T . N E T	253	33.3
229E	179	. S A F V G A L P K T V R E F V I S R T G	199	14.3
NL63	249	S . L Y Q P . R . T C L W P V . . L K . .	269	42.9
OC43	251	S H Y Y V M P L T C I . R R N I . F T L E	271	28.6

S1011

				Score
SARS-CoV-2	1011	Q L I R A A E I R A S A N L A A T K	1028	
HKU1	1096	. . S D I S L V K F G . A . . M E .	1113	44.4
229E	894	T . T K Y T . V . . . R Q . . Q Q .	911	61.1
NL63	1077	V . N K Y T . V . S . R R . . Q Q .	1094	50.0
OC43	1104	. . S D S T L V K F . . A Q . M E .	1121	44.4

S326

				Score
SARS-CoV-2	326	I V R F P N I T N L C P F G E -	340	
HKU1	317	V H . R I P D L P D . D I D K .	331	20.0
229E	289	. . S L . V Y H K - H T . I V L	303	20.0
OC43	333	V Y . R K P D L P N . N I E A .	347	20.0

S1151

				Score
SARS-CoV-2	1151	E L D K Y F K N H T S P D V D	1165	
HKU1	1237	. . S H W . . . Q . . I A P N	1251	60.0
229E	1037	T . Q E L S Y K L P N Y T . P	1051	13.3
NL63	1227	N . P . . V . P N F D L T P F	1241	26.7
OC43	1245	. . . Q W . . . Q . . V A P .	1259	66.7

S336

				Score
SARS-CoV-2	336	C P F G E V F N A T R F A S V	350	
HKU1	327	. D I D K W L . N F N V P . P	341	20.0
OC43	343	. N I E A W L . D K S V P . P	357	20.0

ORF8 43

				Score
SARS-CoV-2	43	S K W Y I R V G A R K S A P L	57	
HKU1	76	K E Y P L L T . Y P L L K Q K	90	20.0

S451

				Score
SARS-CoV-2	451	Y L Y R L F R K S N L K P F E R D I	468	
HKU1	448	. G F N N . N L . S H S V V Y S R Y	465	22.2
NL63	539	S S W H I Y L . . G T C . . S F S K	556	44.4
OC43	462	K R F G F I E D . V F . . Q P A G V	479	27.8

ORF9 NP 127

				Score
SARS-CoV-2	127	K D G I I W V A T E G A L N T	141	
HKU1	141	L E . V F . . . N H Q . D T S	155	53.3
229E	97	V E . V V . . . V D . . K T E	111	66.7
NL63	95	S . . V V . . . K . . . K T V	109	66.7
OC43	142	I . . V Y . . . S N Q . D V N	156	53.3

Table S5: Summary of T cell clones

Specificity	Clone ID	Donor	Correct specificity (tet or stim)	Cross-reactive tetramer staining	Cross-reactive peptide stimulation	Avidity	Stool lysate stimulation	Bacterial lysate stimulation
S462	1C3	HD1	confirmed					Y
S462	1D8	HD1	confirmed					Y
S462	1E5	HD1	confirmed					Y
S462	1E9	HD1	confirmed					
S462	1E11	HD1	confirmed					Y

S462	1F6	HD1	confirmed					Y
S462	1F7	HD1	confirmed					Y
S462	2C9	HD1	confirmed					Y
S462	2C11	HD1	confirmed					Y
S936	C3	HD1	confirmed	Y	Y		Y	Y
S936	C9	HD1	confirmed	Y	Y	Y	Y	Y
S936	F8	HD1	confirmed					
S936	G6	HD1	confirmed					
S936	H6	HD1	confirmed	Y	Y		Y	Y
S936	1C11	HD1	confirmed	Y	Y		Y	
S936	1E7	HD1	confirmed	Y	Y		Y	Y
S936	1F2-2	HD1	confirmed	Y	Y		Y	Y
S936	1G4	HD1	confirmed					
S936	2E2	HD1	confirmed	Y	Y		Y	Y
S936	2E9	HD1	confirmed	Y	Y		Y	Y
S936	2F8	HD1	confirmed	Y	Y		Y	Y
S936	1B8	HD10	confirmed	Y	Y	Y	Y	Y
S936	1C5	HD10	confirmed	Y	Y	Y	Y	Y
S936	1D3	HD10	no					
S936	1F2-1	HD10	confirmed	Y	Y		Y	Y
S936	2E4	HD10	confirmed					
ORF8	2C3	HD10	confirmed					Y
ORF8	2D11	HD10	confirmed					Y
ORF9	2B3	HD5	no					
ORF9	2B8	HD5	confirmed					Y
ORF9	2C6	HD5	confirmed					Y
ORF9	2D3	HD5	confirmed					Y
ORF9	2D5	HD5	confirmed					Y

Y: the indicated experiment was performed.

Table S6: Surface antibody staining panels

Specificity	Fluorochrome	Clone ID	Flow cytometry
CD3	BV785	UCHT1	conventional flow
CD45RO	BV605	UCHL1	conventional flow
ICOS	Alexa Fluor 700	C398.4A	conventional flow
CD3	BUV395	UCHT1	Spectral
CD45RO	BV570	UCHL1	Spectral
CD95	BV711	DX2	Spectral
CD49d	BV750	9F10	Spectral
CD103	BV785	Ber-ACT8	Spectral
Integrin β1	Superbright 600	TS2/16	Spectral
CCR2	BUV737	LS132	Spectral
CCR6	PerCP-efluor 710	R6H1	Spectral
CCR10	PE	563656	Spectral
CLA	Pacific Blue	HECA-452	Spectral
GPR15	BV421	SA302A10	Spectral
CXCR3	Alexa Fluor 647	G025H7	Spectral
CXCR4	BUV563	12G5	Spectral
CXCR5	BB515	RF852	Spectral
PD-1	Alexa Fluor 700	EH12.2H7	Spectral
CD39	BV480	TU66	Spectral

CD127	Alexa Fluor 532	eBioRDR5	Spectral
CD25	APC-Fire 810	M-A251	Spectral
CD38	PE-Cy5	HIT2	Spectral
ICOS	BUV496	DX29	Spectral
CD4	BV510	SK3	Spectral / conventional flow
CCR7	BV650	G043H7	Spectral / conventional flow
Integrin β7	PerCPcy5.5	FIB504	Spectral / conventional flow
CCR9	APC	L053E8	Spectral / conventional flow
CD11b	APC-Cy7	ICRF44	Spectral / conventional flow
CD19	APC-Cy7	HIB19	Spectral / conventional flow

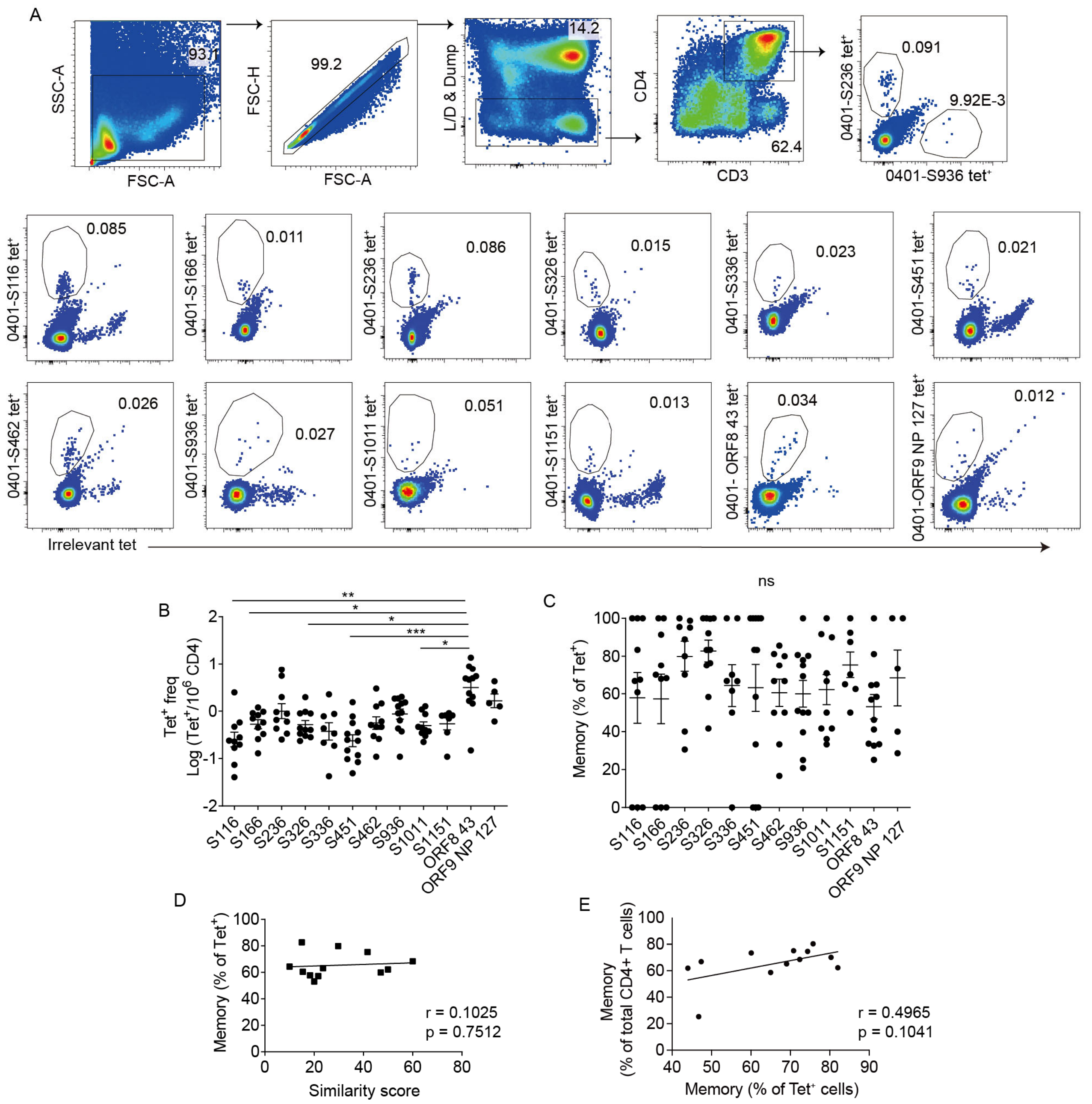

Figure S1: CD4⁺ T cells from unexposed individuals recognized distinct SARS-CoV-2 epitopes
(A) Plots show the gating strategy for identifying tetramer⁺ cells shown in Fig. 1A and the representative staining for each SARS-CoV-2 tetramer. (B-C) Plots show the frequency (B) or memory phenotype (C) of CD4⁺ T cells that recognized the indicated epitope. Each symbol represents data from one donor repeated an average of 2.04 times (±0.07). (D) Relationship between the proportion of memory cells within a tetramer⁺ population and conservation of sequences with common circulating strains as indicated by the similarity score. Each symbol represents one T cell specificity and combines data from different donors. (E) Correlation between the proportion of memory cells in total CD4⁺ T cells and tetramer⁺ cells. Distinct tetramer⁺ populations from the same donor are combined and represented as an average (n = 12). For (B) and (C), Welch' ANOVA was used with p-values for pairwise comparisons computed using Dunnett's T3 procedure. Pearson correlation was computed for (D) and Spearman correlation was computed for (E). Mean ± SEM. * $p < 0.05$, ** $p < 0.01$, *** $p < 0.001$.

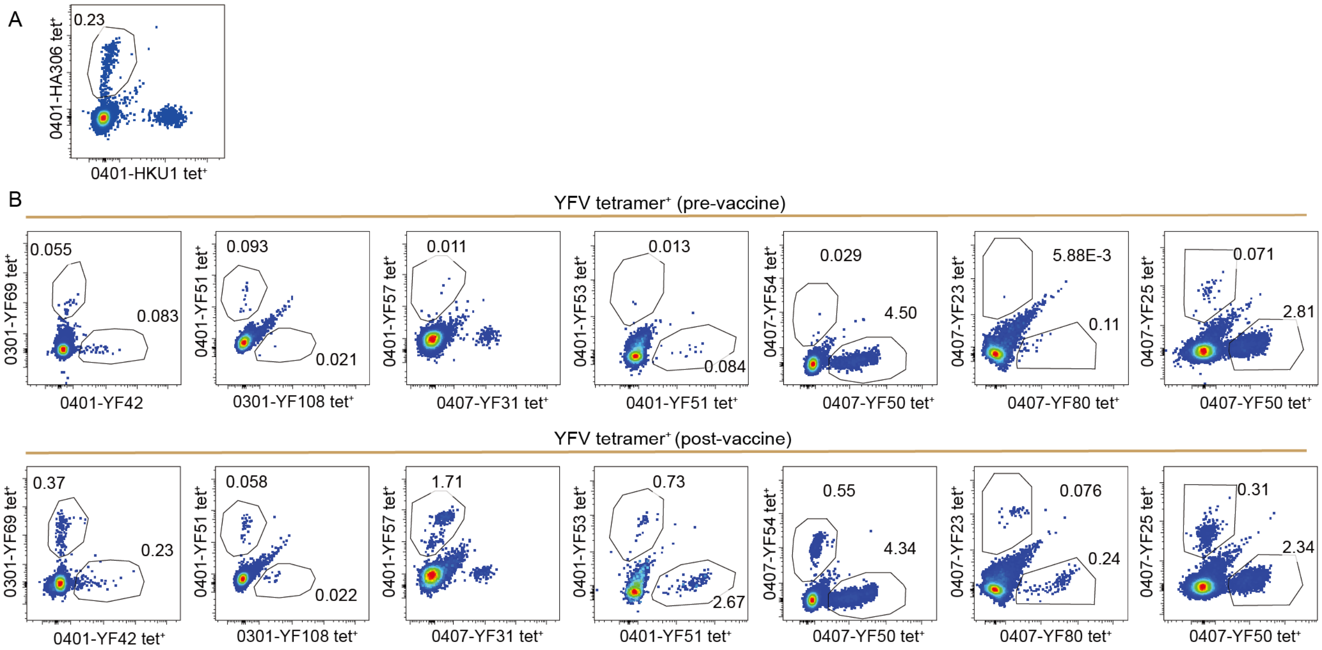

Figure S2: Influenza and YFV-specific CD4+ T cells
(A) Plot show representative staining of HA306 tetramer positive cells. (B) YFV-specific T cells before (pre) or after (post) YFV vaccination from a previously published dataset (*4*). Tetramer gates identify populations shown in Fig. 1D.

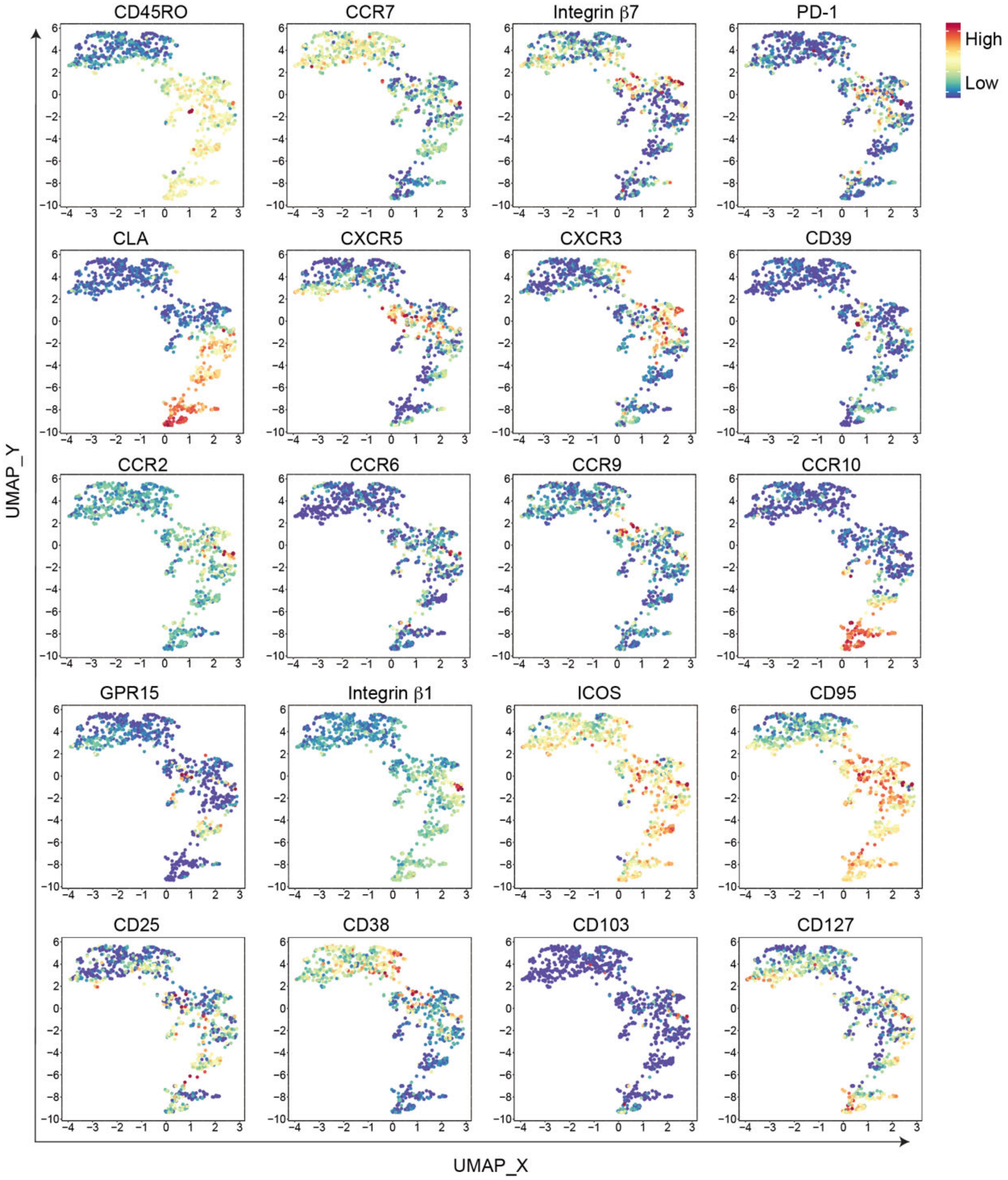

Figure S3: High-dimensional phenotypic analyses of pre-immune SARS-CoV-2-specific T cells
UMAPs display individual markers as indicated. Markers used to select input cells were excluded. Plots combine SARS-CoV-2 tetramer$^+$ cells from six individuals.

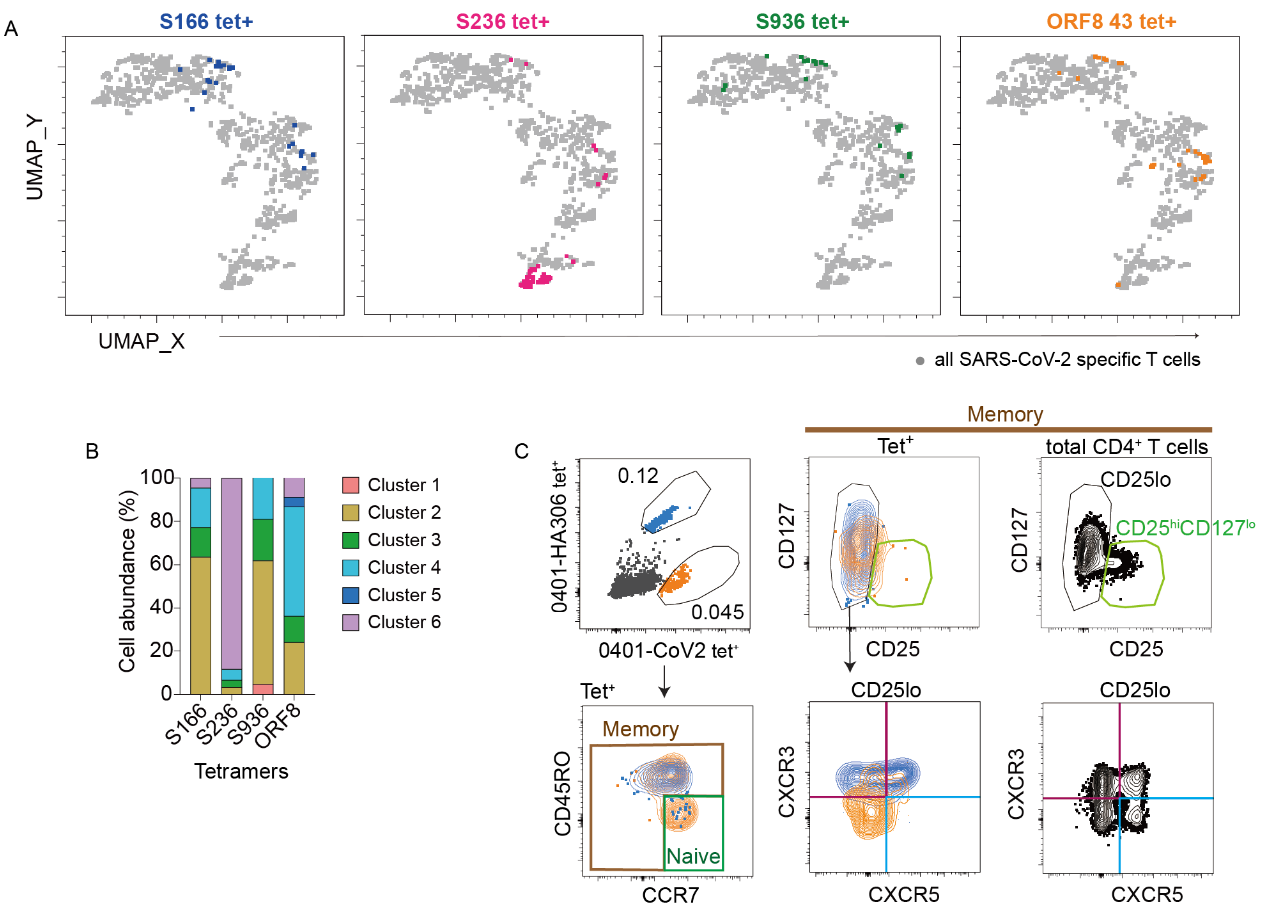

Figure S4: Phenotypic analyses of individual SARS-CoV-2-specific populations
(A) Tetramer^{+} cells of the indicated specificity are projected onto an UMAP that includes all SARS-CoV-2-specific T cells. (B) Bar-graph shows the relative cluster abundance by antigen-specificity. (C) Representative plots show the gating path to identify SARS-CoV-2 and HA306 tetramer-labeled T cells and phenotypic subsets. Manual gating was performed based on the expression of individual phenotypic marker on total CD4^{+} T cells and applied onto tetramer^{+} populations.

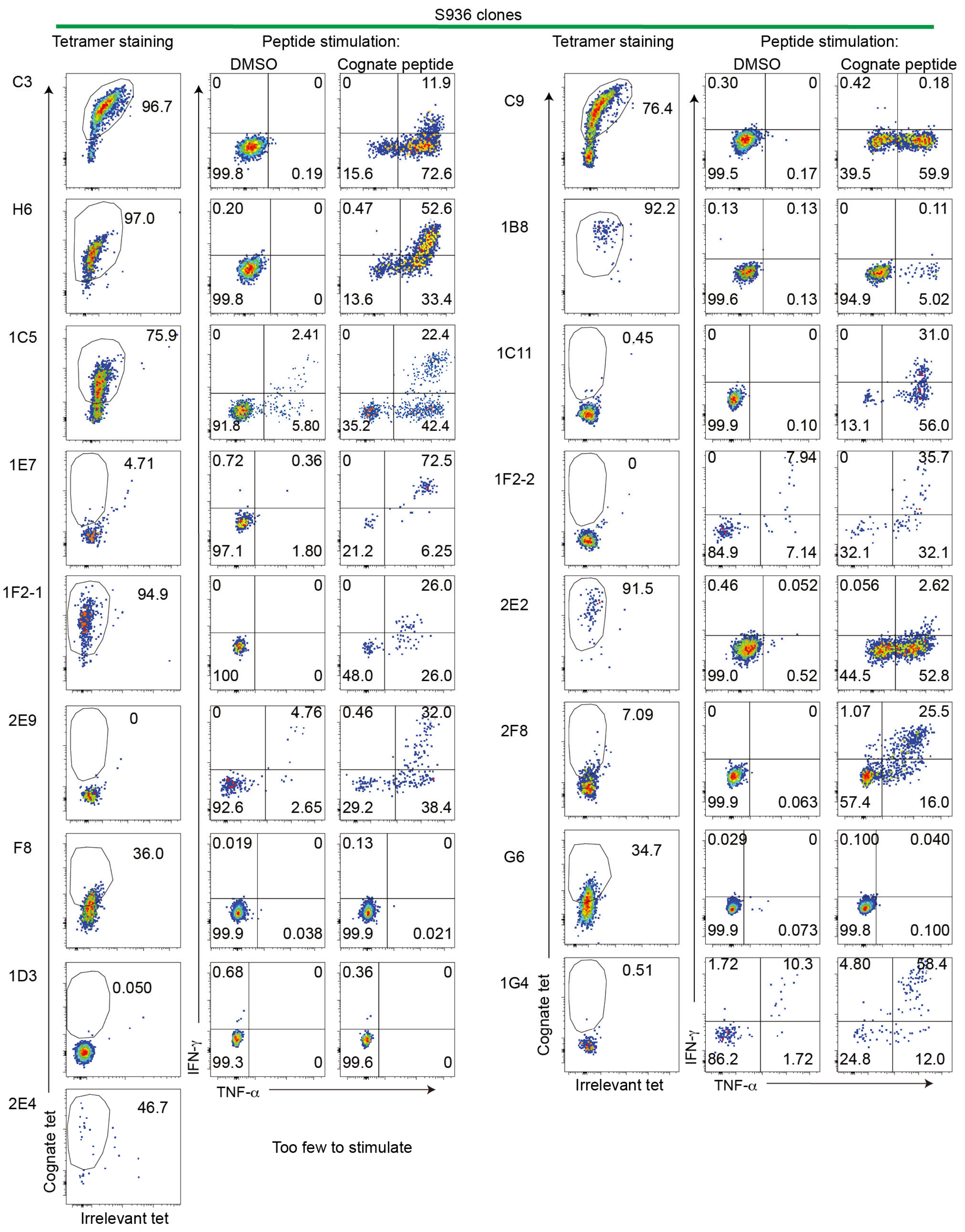

Figure S5: Confirmation of S936-specific T cell clones generated from single tetramer⁺ T cells
Representative plots of the indicated S936 T cell clones. Left: tetramer staining was performed on clones generated from sorted single S936 tetramer-labeled T cells after expansion for 2-3 weeks in culture. Right: T cell clones were incubated with DC treated with vehicle control or S936 peptide and assayed for intracellular cytokine production. Each clone was validated 1-2 times.

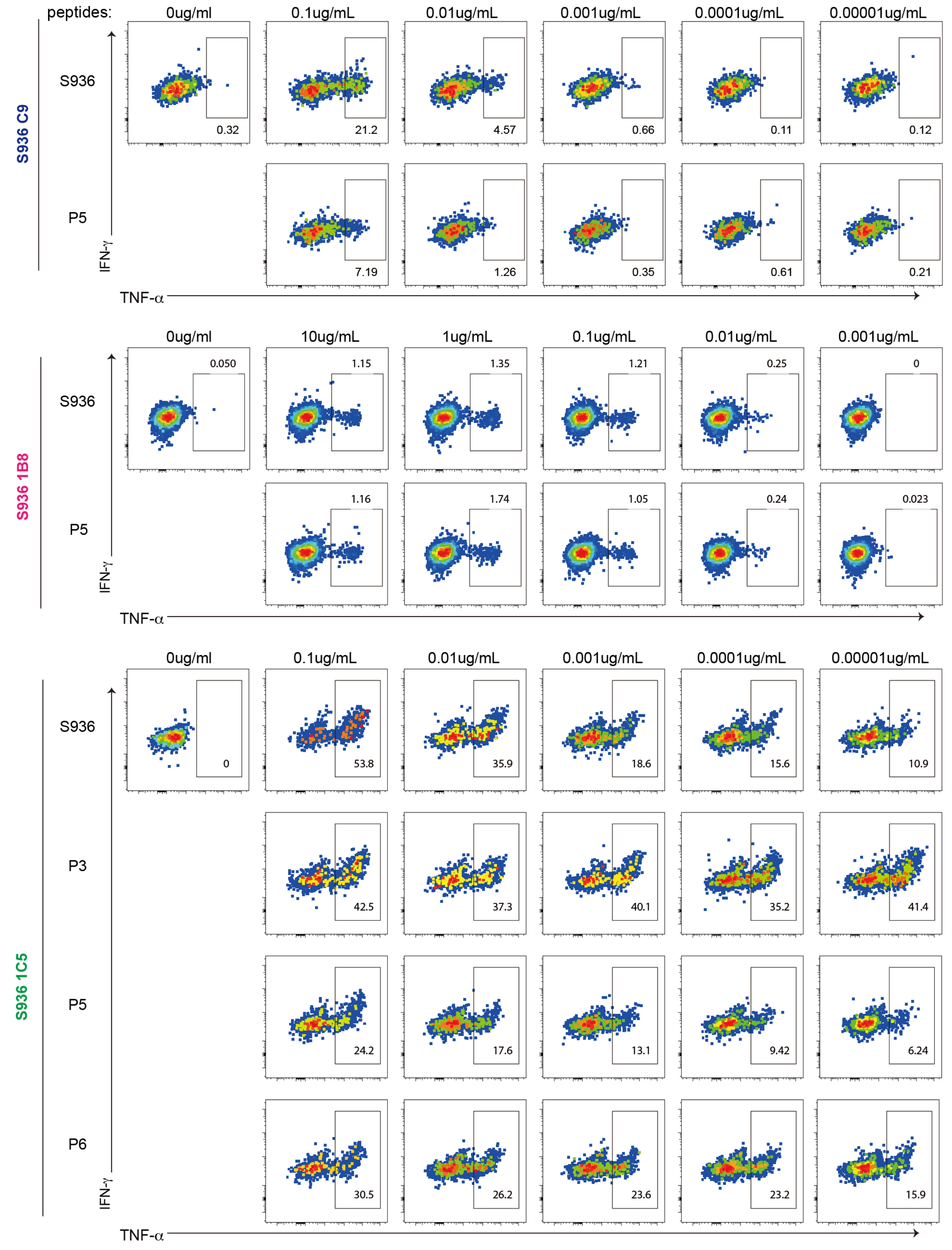

Figure S6: Functional avidity of S936-specific T cell clones in response to cognate and cross-reactive peptides

Plots show representative cytokine response by the indicated clones to decreasing concentrations of the cognate S936 peptide or the bacterial peptide P5. S936 1C5 clone was also stimulated with P3 and P6.

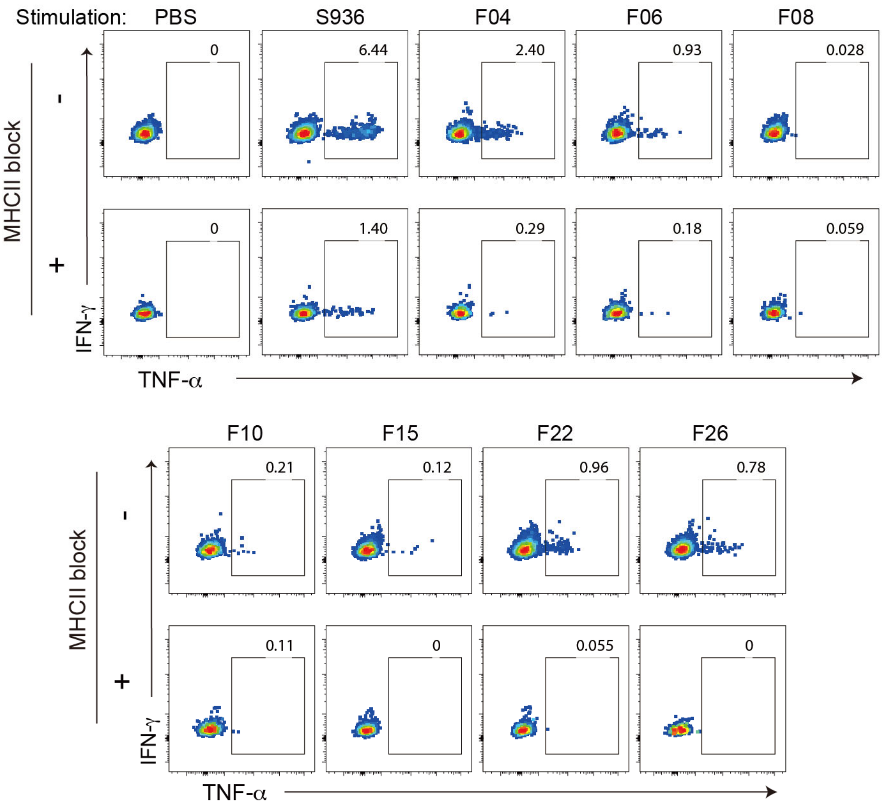

Figure S7: T cell response to fecal lysates with or without MHC inhibition
S936 1F2-1 clone was stimulated for 5 hours with DC treated with PBS, fecal lysates, or the cognate S936 peptide. Plots show representative cytokine response. T cell stimulation was carried out in the presence or absence of MHC II blocking antibodies as indicated.

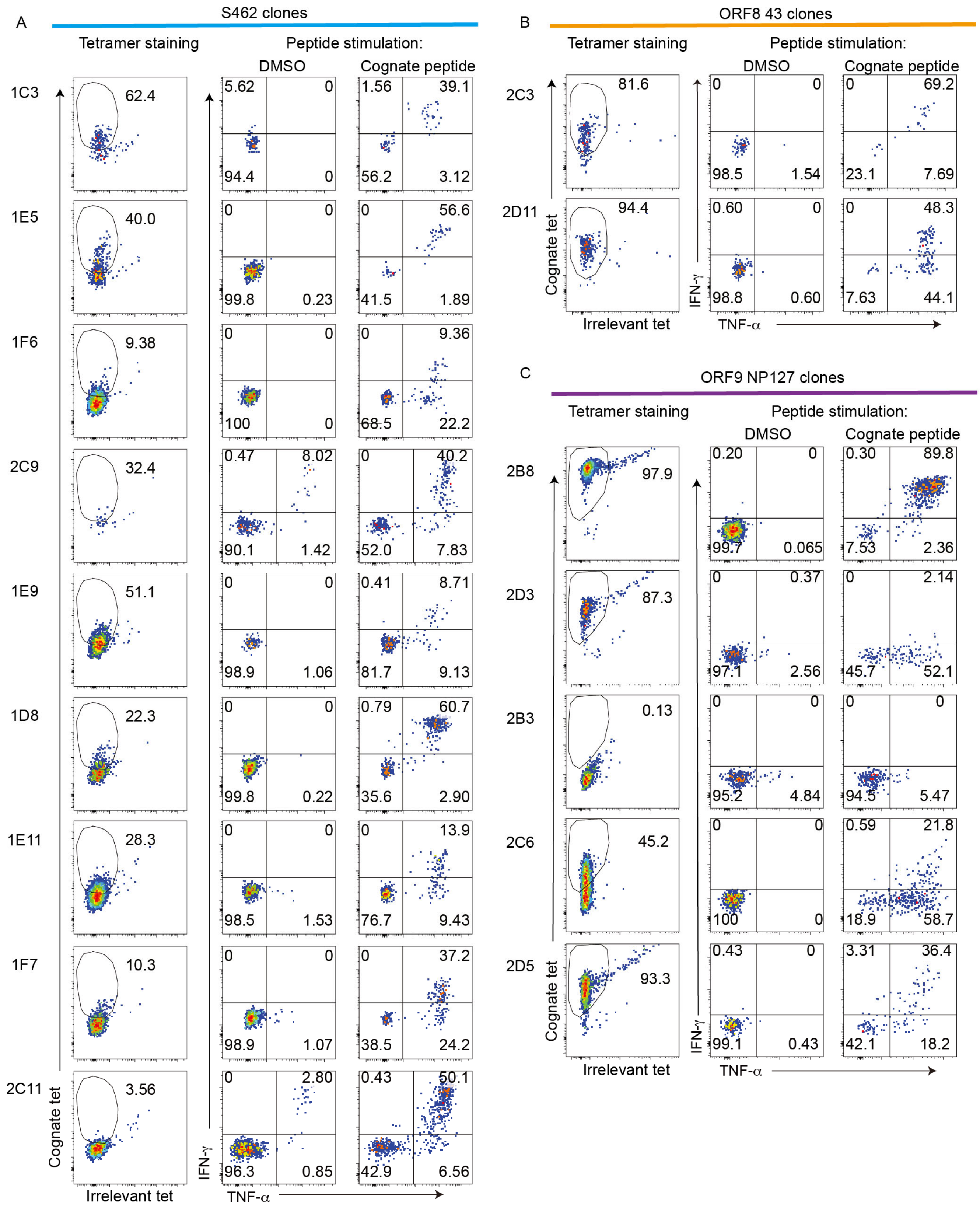

Figure S8: Confirmation of S462, ORF8, and ORF9-specific T cell clones generated from single tetramer⁺ T cells

Representative plots of the indicated clones. Left: tetramer staining was performed on clones generated from sorted single tetramer-labeled T cells after expansion for 2-3 weeks in culture. Right: T cell clones were incubated with DC treated with vehicle control or the cognate peptide and assayed for intracellular cytokine production. Each clone was validated 1-2 times.

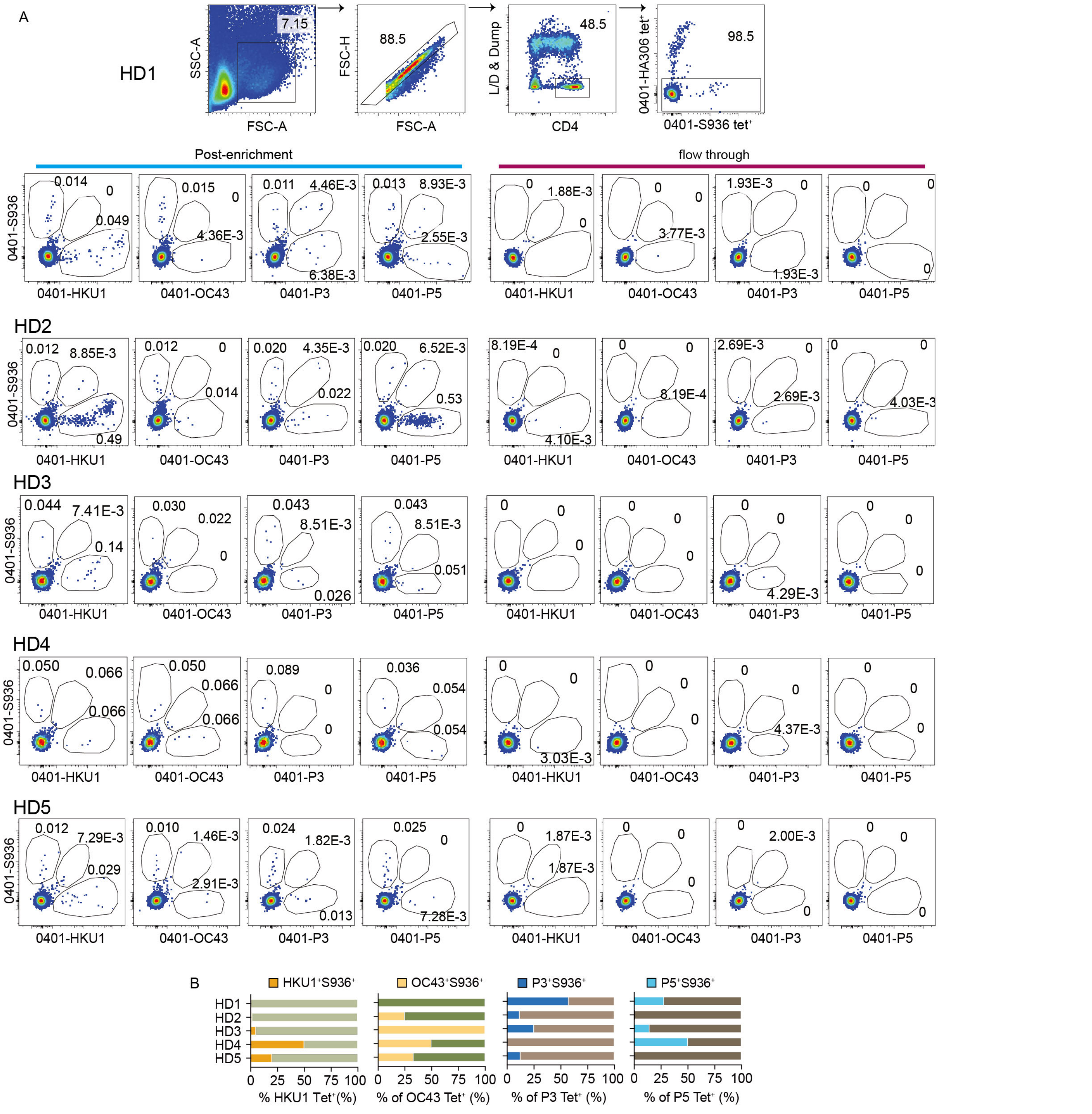

Figure S9: Gating strategy for identifying cross-reactive T cells

(A) Representative plots show the gating strategy for identifying tetramer⁺ cells in the post-enrichment fraction after magnetic column enrichment (left). Gates were set to minimize the detection of cells in the flow-through fraction (right). Tetramer staining was performed on cells from five unexposed individuals (HD1-5). (B) Bar-graphs show the frequency of T cells that cross-reacted with HKU1 or OC43 sequences as a percentage of total HKU1, OC43, P3, or P5-specific T cells. Each bar represents data from one individual, repeated 1-2 times depending on sample availability.

References and notes

1. J. Mateus, A. Grifoni, A. Tarke, J. Sidney, S. I. Ramirez, J. M. Dan, Z. C. Burger, S. A. Rawlings, D. M. Smith, E. Phillips, S. Mallal, M. Lammers, P. Rubiro, L. Quiambao, A. Sutherland, E. D. Yu, R. da Silva Antunes, J. Greenbaum, A. Frazier, A. J. Markmann, L. Premkumar, A. de Silva, B. Peters, S. Crotty, A. Sette, D. Weiskopf, Selective and cross-reactive SARS-CoV-2 T cell epitopes in unexposed humans. *Science*, (2020).
2. Y. Peng, A. J. Mentzer, G. Liu, X. Yao, Z. Yin, D. Dong, W. Dejnirattisai, T. Rostron, P. Supasa, C. Liu, C. Lopez-Camacho, J. Slon-Campos, Y. Zhao, D. I. Stuart, G. C. Paesen, J. M. Grimes, A. A. Antson, O. W. Bayfield, D. Hawkins, D. S. Ker, B. Wang, L. Turtle, K. Subramaniam, P. Thomson, P. Zhang, C. Dold, J. Ratcliff, P. Simmonds, T. de Silva, P. Sopp, D. Wellington, U. Rajapaksa, Y. L. Chen, M. Salio, G. Napolitani, W. Paes, P. Borrow, B. M. Kessler, J. W. Fry, N. F. Schwabe, M. G. Semple, J. K. Baillie, S. C. Moore, P. J. M. Openshaw, M. A. Ansari, S. Dunachie, E. Barnes, J. Frater, G. Kerr, P. Goulder, T. Lockett, R. Levin, Y. Zhang, R. Jing, L. P. Ho, T. c. C. Oxford Immunology Network Covid-19 Response, I. C. Investigators, R. J. Cornall, C. P. Conlon, P. Klenerman, G. R. Screaton, J. Mongkolsapaya, A. McMichael, J. C. Knight, G. Ogg, T. Dong, Broad and strong memory CD4(+) and CD8(+) T cells induced by SARS-CoV-2 in UK convalescent individuals following COVID-19. *Nat Immunol* **21**, 1336-1345 (2020).
3. A. Nelde, T. Bilich, J. S. Heitmann, Y. Maringer, H. R. Salih, M. Roerden, M. Lubke, J. Bauer, J. Rieth, M. Wacker, A. Peter, S. Horber, B. Traenkle, P. D. Kaiser, U. Rothbauer, M. Becker, D. Junker, G. Krause, M. Strengert, N. Schneiderhan-Marra, M. F. Templin, T. O. Joos, D. J. Kowalewski, V. Stos-Zweifel, M. Fehr, A. Rabsteyn, V. Mirakaj, J. Karbach, E. Jager, M. Graf, L. C. Gruber, D. Rachfalski, B. Preuss, I. Hagelstein, M. Marklin, T. Bakchoul, C. Gouttefangeas, O. Kohlbacher, R. Klein, S. Stevanovic, H. G. Rammensee, J. S. Walz, SARS-CoV-2-derived peptides define heterologous and COVID-19-induced T cell recognition. *Nat Immunol*, (2020).
4. Y. G. Pan, B. Aiamkitsumrit, L. Bartolo, Y. Wang, C. Lavery, A. Marc, P. V. Holec, C. G. Rappazzo, T. Eilola, P. A. Gimotty, S. E. Hensley, R. Antia, V. I. Zarnitsyna, M. E. Birnbaum, L. F. Su, Vaccination reshapes the virus-specific T cell repertoire in unexposed adults. *Immunity*, (2021).
